# Modulating Liquid–Liquid Phase Separation of Nck Adaptor Protein against Enteropathogenic *Escherichia coli* Infection

**DOI:** 10.1021/acscentsci.3c01068

**Published:** 2023-12-14

**Authors:** Min Liu, Chunjian Wu, Rui Wang, Jiaming Qiu, Zhentao She, Jianan Qu, Jiang Xia

**Affiliations:** † Department of Chemistry and Center for Cell & Developmental Biology, The Chinese University of Hong Kong, Shatin, Hong Kong SAR, China; ‡ Pingshan Translational Medicine Center, Shenzhen Bay Laboratory, Shenzhen 518118, China; § Departments of Electronic and Computer Engineering, Center of Systems Biology and Human Health, School of Science and Institute for Advanced Study, Hong Kong University of Science and Technology, Clear Water Bay, Kowloon, Hong Kong SAR, China

## Abstract

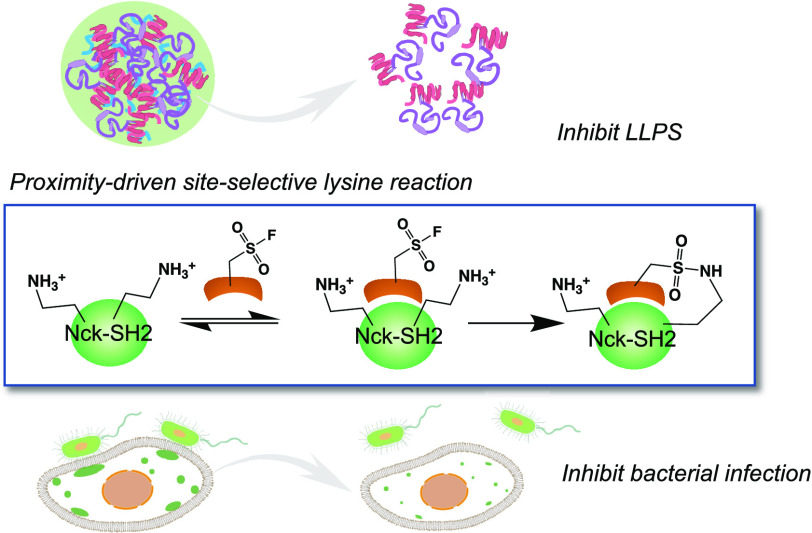

Signaling proteins often form biomolecular condensates through liquid–liquid phase separation (LLPS) during intracellular signal transduction. Modulating the LLPS property of intracellular protein condensates will redirect intracellular signals and provide a potential way to regulate cellular physiology. Phosphorylation of multiple tyrosine residues of the transmembrane receptor nephrin is known to drive the LLPS of the adaptor protein Nck and neuronal Wiskott–Aldrich Syndrome protein (N-WASP) and form the Nck signaling complex. Phosphorylation of the translocated intimin receptor (Tir) in the host cell may recruit this enteropathogenic *Escherichia coli* (EPEC) virulence factor to the Nck signaling complex and lead to the entry of EPEC into the intestine cell. In this work, we first identified a phosphotyrosine (pY)-containing peptide **3pY** based on the sequence similarity of nephrin and Tir; **3pY** promoted the LLPS of Nck and N-WASP, mimicking the role of phosphorylated nephrin. Next, we designed a covalent blocker of Nck, peptide **p1** based on the selected pY peptides, which site-selectively reacted with the SH2 domain of Nck (Nck-SH2) at Lys331 through a proximity-induced reaction. The covalent reaction of **p1** with Nck blocked the protein binding site of Nck-SH2 and disintegrated the **3pY**/Nck/N-WASP condensates. In the presence of membrane-translocating peptide L17E, **p1** entered Caco-2 cells in the cytosol, reduced the number of Nck puncta, and rendered Caco-2 cells resistant to EPEC infection. Site-selective covalent blockage of Nck thereby disintegrates intracellular Nck condensates, inhibits actin reorganization, and shuts down the entrance pathway of EPEC. This work showcases the promotion or inhibition of protein phase separation by synthetic peptides and the use of reactive peptides as LLPS disruptors and signal modulators.

## Abstract 2

A designed peptide selectively reacts with a single lysine residue on Nck, disrupts phase-separated signaling coacervates, shuts down an intracellular signal, and inhibits *Escherichia coli* infection.

## 1 Introduction

**Figure 1:**
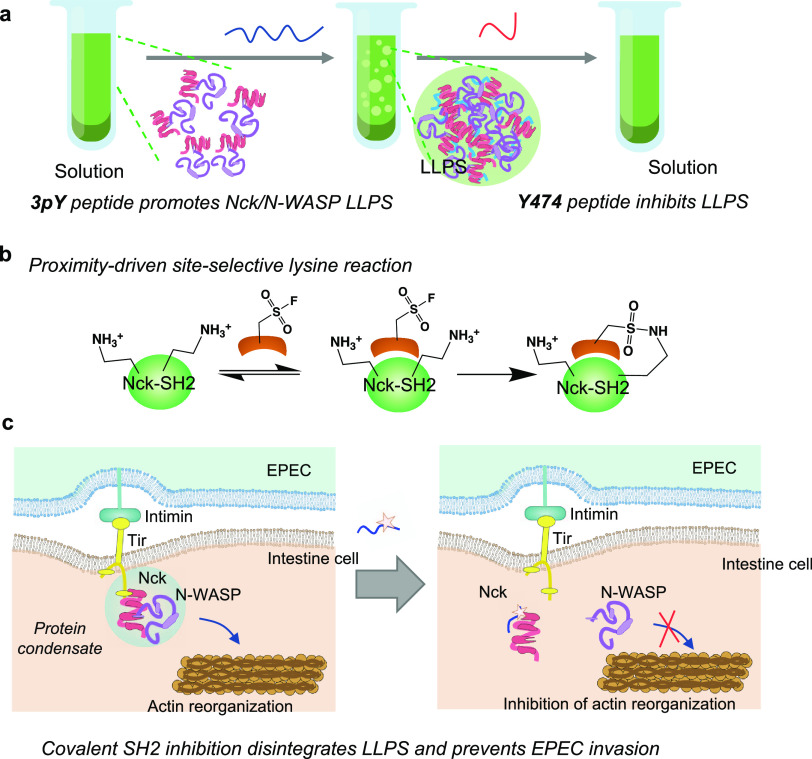
Schematic illustration summarizing the main discoveries of this work. (a) Schematic illustration showing that a trimeric pY-containing peptide promotes LLPS of Nck and N-WASP, and covalent and noncovalent Nck-SH2 blockers reverse the coacervate formation. (b) Proximity-induced lysine reaction of Nck-SH2 yields covalent SH2 blockers with a selected lysine. (c) Covalent and noncovalent Nck-SH2 inhibitors disintegrate Nck coacervates in cells and prevent EPEC invasion.

Severe or persistent diarrhea is the second leading cause of death in children younger than 5 years old, accounting for 1.3 million deaths annually, especially in low- and middle-income countries.^1−3^ Among all the diarrheagenic *Escherichia coli* pathotypes that cause diarrhea, enteropathogenic *Escherichia coli* (EPEC) is a major pathotype highly prevalent in the community and hospitals.^4−7^ The emerging multidrug-resistant strains also have drastically increased the difficulty of treating EPEC-caused infections.^8−10^ The mechanism of EPEC infection centers around signal transduction of the adaptor protein Nck. An EPEC bacterium first latches to the surface of an intestinal cell and injects one of the virulence factors, the translocated intimin receptor (Tir), into the cell. Tir is encoded by *espE* located on the locus of enterocyte effacement (LEE) pathogenicity island in EPEC strains. These steps result in an attaching and effacing (A/E) lesion between the EPEC bacterium and the intestine cell.^6,11^ Phosphorylation of the Tir protein at Tyr 474 induces its binding with the SH2 domain of Nck (Nck-SH2), which subsequently recruits Nck-binding proteins such as WIP and N-WASP and activates the Arp2/3 complex-dependent actin polymerization pathway in the host cells.^12^ Actin polymerization forms a pedestal underneath the bacterium and leads to the invasion of EPEC and other microbes, such as vaccinia^13,14^ and other vertebrate poxviruses.^15^ Nck proteins (Nck1 and Nck2, also called Nckβ and Grb4) contain four domains in tandem, including one SH2 domain and three SH3 domains. Nck-SH2 is known to bind to the phosphorylated peptide sequence on the Tir protein in EPEC, the viral membrane protein A36, and nephrin.^12,16,17^ Because of the high degree of sequence homology of Nck1 and Nck2, Nck1 (for simplicity, Nck hereinafter) was chosen in this work. Due to the essential role of the interaction between Tir and Nck-SH2 in EPEC infection, we envision that a peptide that blocks the protein-binding site of the Nck-SH2 domain will inhibit EPEC-mediated pedestal formation, which will block the entrance of EPEC and guard the cells against EPEC infection (Figure 1).

Biological molecules, such as proteins or nucleic acids, form biomolecular condensates or coacervates and function as membrane-less organelles in eukaryotic cells.^18,19^ The condensates are highly concentrated micron-sized liquid droplets formed by liquid–liquid phase separation (LLPS) driven by physical interactions.^20^ The changes in physical states of biomolecules are associated with the occurrence and prevention of some diseases, such as protein aggregates involved in neurodegenerative diseases, protein accumulation^21^ or transcription abnormal in cancer,^22^ and viral replication condensates in SARS-CoV-2 infection.^23^ Rosen and co-workers discovered that the adaptor protein Nck serves as a platform for a multivalent interaction and forms LLPS coacervates with the actin-regulatory protein called neural Wiskott–Aldrich syndrome protein (N-WASP) and phosphorylated nephrin.^24−29^ Nck phase transition is associated with the activity of an actin nucleation factor, the Arp2/3 complex, and is governed by the degree of nephrin phosphorylation. However, the role of Nck-mediated LLPS in infectious diseases and whether LLPS can derive druggable targets for treating bacterial infections remain largely untapped.

Because Nck-SH2 binds to phosphorylated Tir, and this interaction drives EPEC invasion, we reason that blocking Nck-SH2 will disintegrate the Nck signaling complex and shut down the Nck-mediated actin rearrangement. However, the SH2 domains in mammalian cells are highly promiscuous, so selective blocking of the Nck-SH2 domain among ∼120 SH2 domains in the proteome is difficult.^30^ Because the phosphorylated tyrosine (pY) residue provides approximately half of the total binding energy in the pY-peptide–SH2 complex, it is challenging to design high-affinity and high-selectivity blockers specifically for the Nck-SH2 domain while sparing other SH2 domains or pY-binding proteins in the cytoplasm. In previous work, we developed proximity-driven cysteine-selective reactions between pY-containing peptides with SH2 domains using the reaction between an α-chloroacetyl group and a cysteine. This allowed us to differentiate different SH2 domains and achieve high-affinity covalent inhibition of specific SH2 domains selectively.^30^ The site-selective cysteine reactions were also brought inside cells, leading to intracellular domain-specific irreversible inhibition of a PDZ domain and an SH3 domain and effective blockage of signal transduction inside cells and even in animals.^31,32^ These covalently reactive peptides may be precursors of covalent drugs.^33−36^ Here, we first validate that Nck forms phase-separated condensates with N-WASP and a pY peptide trimer (Figure 1a). Then, a structure-guided design leads to a reactive peptide that can covalently react with the Nck-SH2 domain at a selected lysine residue to interfere with the Nck/N-WASP LLPS (Figure 1b). We also report the intracellular delivery of this reactive peptide, the disintegration of Nck coacervates, and the blockage of EPEC infection in intestine cells (Figure 1c).

## 2 Results and Discussion

### A Peptide Promoter of Nck Phase Separation *in Vitro*

**Figure 2:**
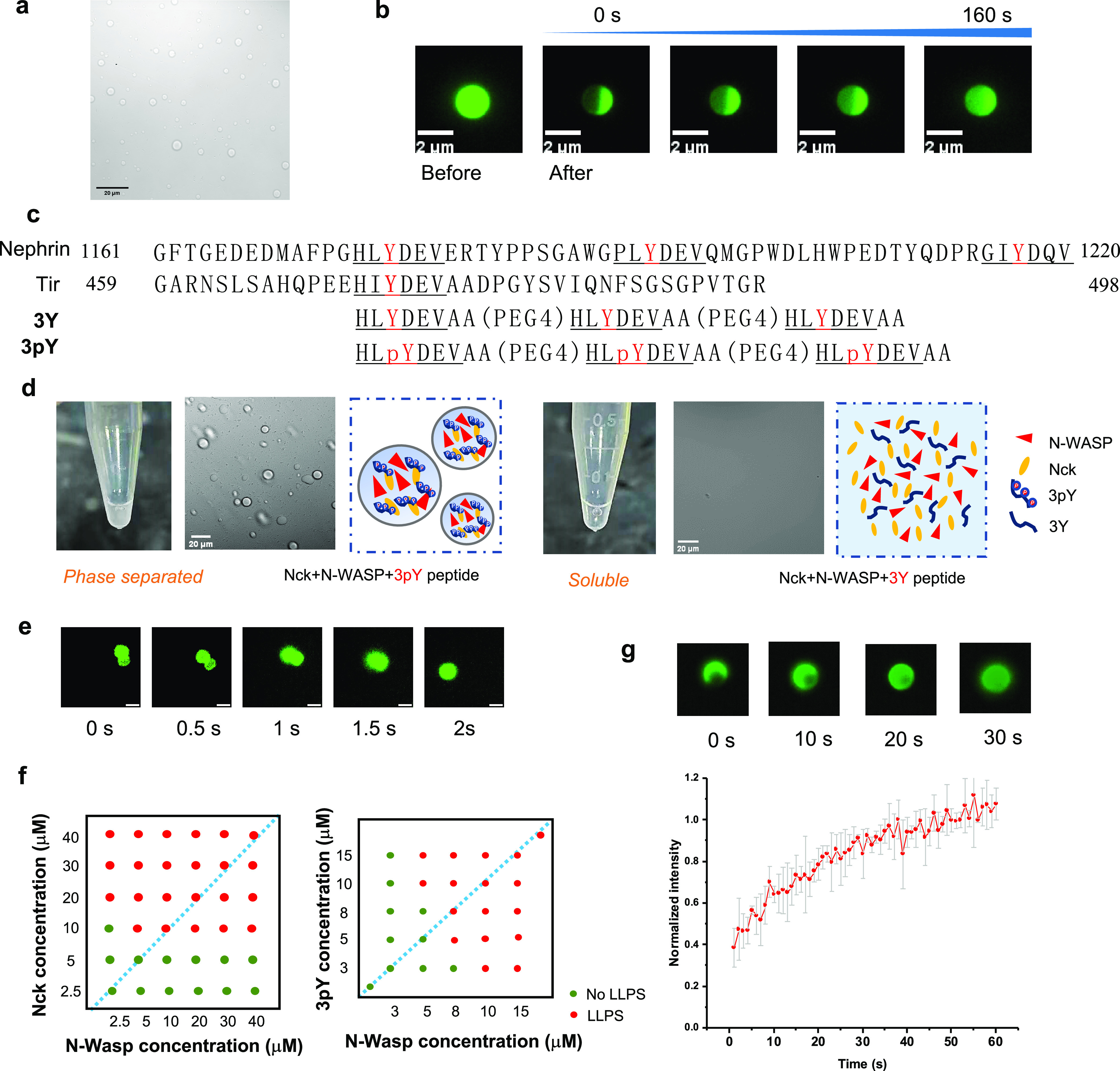
Phosphopeptide 3pY promotes Nck/N-WASP phase separation. (a) Microscopic image showing Nck and N-WASP form droplets at high concentrations (Nck, 40 μM; N-WASP, 60 μM). (b) Recovery of fluorescence after bleaching, suggesting the Nck/N-WASP droplets have liquid-like properties. (c) Design of a **3pY** peptide based on the sequence alignment of Tir and nephrin. The Nck-SH2 binding sequences are underscored, and the Tyr sites that are potentially phosphorylated are marked in red. (d) Macroscopic and microscopic images showing that **3pY** promoted the microdroplet formation of Nck/N-WASP, whereas **3Y** did not. Peptides, 10 μM; Nck, 20 μM; N-WASP, 10 μM. Scale bar: 20 μm. (e) Phase diagrams of protein condensate formation at a fixed **3pY** concentration of 10 μM (left) and a fixed Nck concentration of 20 μM (right). (f) Time-lapse microscopy images showing the fusion of two microdroplets. Scale bar: 2 μm. (g) Recovery of fluorescence after bleaching suggesting Nck/N-WASP/**3pY** droplets have liquid-like properties. **3pY**, 10 μM; Nck, 20 μM; N-WASP, 10 μM. Scale bar: 2 μm.

Nck, N-WASP, and p-nephrin are known to undergo liquid–liquid phase separation (LLPS).^24−29^ N-WASP consists of 5 domains: WH1 (WASP-homology 1), BR1 (a highly basic region), GBD (GTPase-binding domain), and proline-rich and VVCA (verprolin homology, central hydrophobic region, acidic region) domains. Here, the GBD-P-VCA tridomain of N-WASP was expressed and purified, and it was called N-WASP thereafter in this work.^27^ In the absence of p-nephrin, Nck and N-WASP formed micrometer-sized microdroplets in aqueous PBS buffer at very high concentrations, i.e., 40 μM of Nck and 60 μM of N-WASP (Figure 2a). The droplets showed liquid-like characteristics based on a fluorescence recovery assay after photobleaching (FRAP) assay (Figure 2b), proving to be coacervates driven by LLPS.

Phosphorylated Tir is known to bind to Nck-SH2. A 12-residue pY-containing Tir peptide EEHIpYDEVAADP was identified as a strong binder to Nck-SH2, and the crystal structure of the complex was reported.^17^ Also, the sequence of nephrin contains three Tyr-containing sequences that are similar to those of the Tir peptide: HLYDEV, PLYDEV, and GIYDQV (the potential phosphorylation sites are underscored), with a hydrophobic residue at the pY-1 site, and negatively charged residues at pY+1 and/or pY+2 (Figure 2c). Therefore, we reason that phosphorylation at these Tyr turns nephrin into p-nephrin, a multivalent Nck-SH2 binder, which drives the LLPS of Nck-SH2 and other binding proteins.^27^ To prove this hypothesis, we designed a trimeric phosphopeptide **3pY** containing three LpYDEV linked through PEG linkers—all with L at pY-1 and D and E at pY+1 and pY+2 sites, to mimic the multivalent phosphorylated nephrin or Tir (Figure 2c). A nonphosphorylated version **3Y** was also synthesized as a control. Adding **3pY** to Nck and N-WASP turned the solution to opalescent, and micron-sized spherical droplets were visible under the microscope at significantly lower protein concentrations (Figure 2d). Under a time-lapse microscope, we observed that the microdroplets could fuse into larger condensates (Figure 2e). A phase diagram revealed that the minimal phase-forming concentrations of Nck, N-WASP, and **3pY** were 10 μM, 5 μM, and 1 μM, respectively (Figure 2f). Next, we fluorescently labeled N-WASP with Cy3 and Nck with Cy5. The Cy3 and Cy5 signals colocalized in the Nck/N-WASP/**3pY** system under the fluorescent microscope (Figure S1 in the Supporting Information). The fluorescence recovery after photobleaching (FRAP) assay showed rapid fluorescence recovery after photobleaching the fluorescent signal within 30 s (Figure 2g). Taken together, these results show that **3pY** promotes the condensation of Nck and N-WASP through the interaction with Nck-SH2 similar to that of p-nephrin, and the condensates formed in the Nck/N-WASP/**3pY** system have liquid-like properties, meeting the properties, of liquid–liquid phase separation (LLPS). We reason that the multivalent feature and two flexible PEG_4_ linkers are essential for **3pY** to promote the LLPS of the Nck/N-WASP system.^27^

### B Peptide Inhibitor of Nck/N-WASP/3pY Coacervation

**Figure 3:**
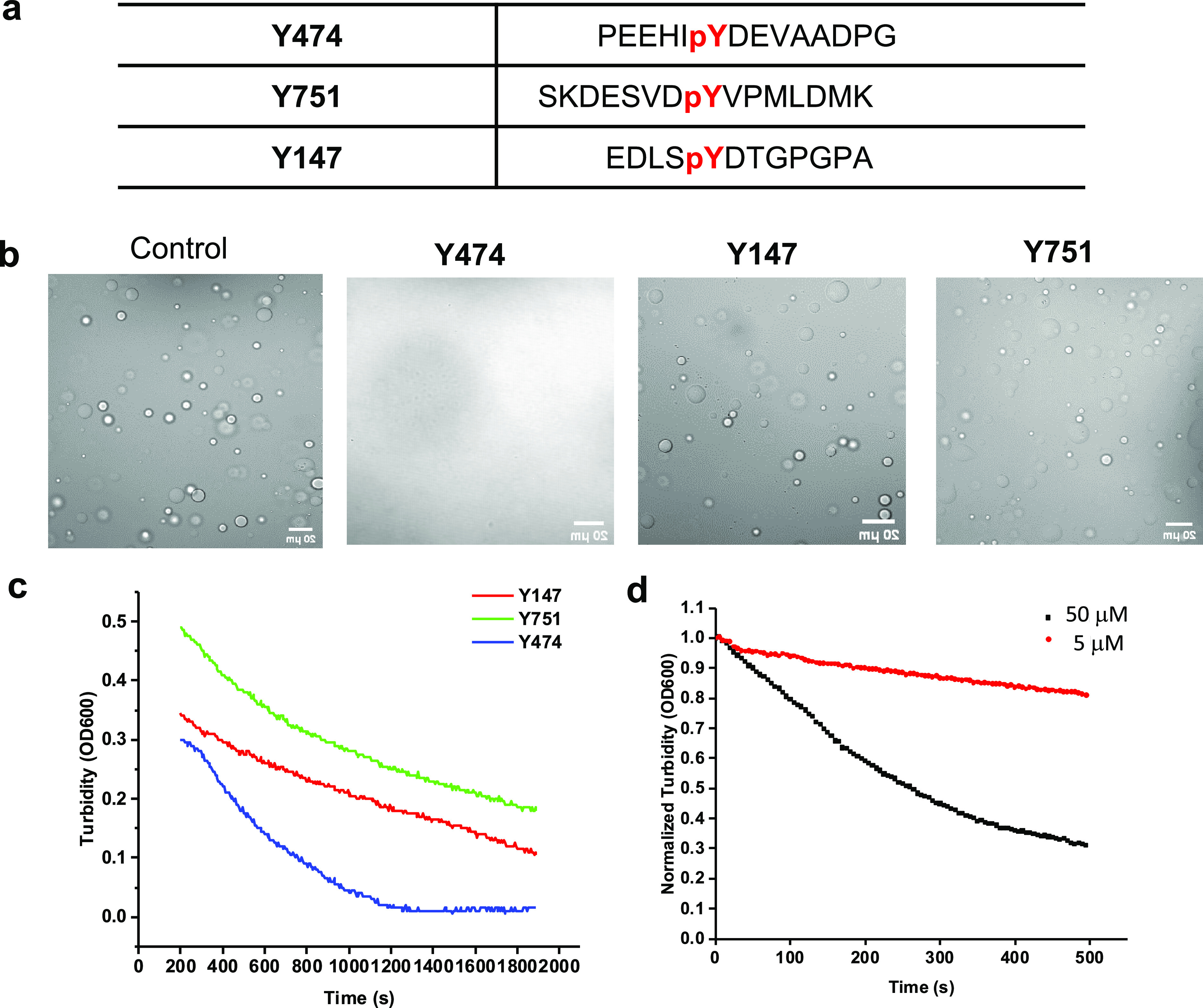
pY peptides disrupt Nck/N-WASP/3pY LLPS. (a) Sequences of synthetic pY peptides. (b) Microscopic images showing the droplet disappearance after adding peptide **Y474** (50 μM) to the Nck/N-WASP/**3pY** system. Nck: 20 μM; N-WASP: 10 μM; **3pY**: 10 μM. Scale bars, 20 μm. (c) Peptide **Y474** rapidly decreases the turbidity of the Nck/N-WASP/**3pY** solution in around 20 min. Nck: 20 μM, N-WASP: 10 μM, **3pY**: 10 μM. (d) 50 μM **Y474** more effectively decreased the turbidity of the Nck/N-WASP/**3pY** solution than 5 μM **Y474**. Nck: 20 μM, N-WASP: 10 μM, **3pY**: 10 μM.

Next, we explored the intervention of Nck/N-WASP/**3pY** LLPS using pY peptides (Figure 3a). Three pY-containing peptides extracted from different Tir sequences were synthesized, namely, peptides **Y474**, **Y751**, and **Y147** (Figure 3b). When peptide **Y474** was added to the Nck/N-WASP/**3pY** system at a high concentration of 50 μM, protein microdroplets disappeared; such an effect was not observed when peptides **Y751** and **Y147** of the same concentration were added to the coacervates (Figure 3c). Using solution turbidity as a measure of coacervate formation, peptide **Y474** caused a rapid decrease in the turbidity of the Nck/N-WASP/**3pY** solution. In contrast, the effect of the other two peptides was significantly less pronounced (Figure 3d). The inhibitory effect of peptide **Y474** was also found to be concentration dependent: 50 μM **Y474** caused more turbidity decrease than 5 μM **y474** (Figure 3e). Also, peptide **Y474** was found to bind with Nck-SH2 with a *K*_D_ of 70 nM, based on the measurement by microscale thermophoresis (MST) (Figure S2 in the Supporting Information). These data show that monovalent Nck-SH2 binding peptides disrupt Nck LLPS at high concentrations by competing with multivalent binders for Nck-SH2 binding sites.

### C Reactive Peptides Based on Proximity-Induced Lysine Reaction

**Figure 4:**
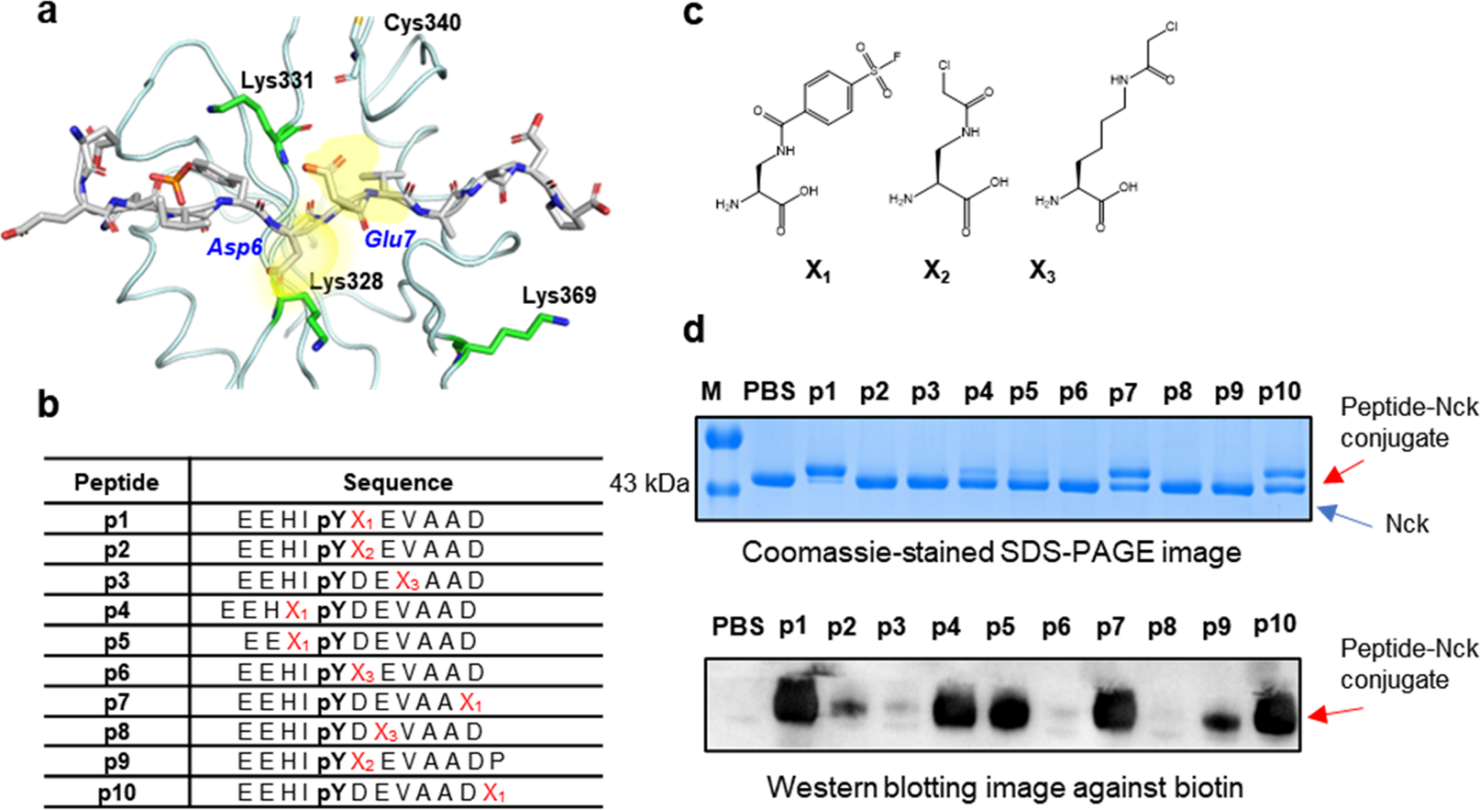
Covalently reactive peptides designed based on a Tir peptide. (a) Crystal structure of Nck-SH2 in complex with a peptide EEHIpYDEVAAD from EPEC protein Tir (PDB: 2CI9). (b) List of synthetic reactive peptides. (c) Unnatural amino acids in the peptides. (d) SDS-PAGE (top) and Western blotting (bottom) analyses showing covalent conjugation of the peptides with Nck. Briefly, biotin-labeled peptides were mixed with recombinant Nck protein at a peptide-to-protein ratio of 5:1 at 37 °C for 1 h before denaturation and resolution by SDS-PAGE.

Although peptide **Y474** can block Nck-mediated LLPS, the requirement of a high concentration of the blocker peptide is hard to achieve inside the cell. We then sought to design covalent, irreversible blockers, anticipating that, at a significantly lower concentration, the covalent blocker can effectively inhibit Nck coacervation in the cell. The cocrystal structure of the Tir peptide and Nck-SH2 (PDB ID: 2CI9) shows that several lysine residues of Nck (including Lys328, Lys331, and Lys369) form a positively charged surface to host negatively charged Glu and Asp at pY+1 and pY+2 sites of the ligand EEHIpYDEVAAD (shown in red) (Figure 4a). The side chains of the Nck lysine residues are pointing toward the residues at pY+1 and pY+2 positions with distances of only 4 to 6 Å. This unique feature gives us an opportunity to design covalent blockers for Nck-SH2 by installing electrophiles at the pY+1 or pY+2 positions to enable a proximal conjugation reaction with one of the lysine residues. Covalent blockage through the characteristic lysine residues will maximize the specificity of the inhibitor toward Nck-SH2. As sulfonyl fluoride has been reported to react with proteinaceous nucleophiles such as lysine, histidine, or cysteine,^34^ we installed a sulfonyl fluoride group on the side chain of diaminopropionic acid, giving an unnatural amino acid X_1_ at different positions such as pY+1 (Asp6) and pY+3 (Val8) of peptide EEHIpYDEVAAD. Besides the amino acid X_1_, we also included α-chloroacetyl-carrying amino acids X_2_ and X_3_, which may also react with proteineous nucleophiles. Electrophile-containing reactive peptides **p1** to **p11** were synthesized with a biotin tag at the N-termini (Figure 4b). After the reactive peptides were incubated with recombinant Nck protein at a 5:1 ratio at 37 °C in PBS for 1 h, the formation of covalently linked Nck–peptide conjugates could be observed, shown as new protein bands with molecular weights higher than that of Nck based on denatured SDS-PAGE and Western blotting analysis against biotin (Figure 4c). The SDS-PAGE gel showed that among all the peptides, **p1** gave the highest cross-linking efficiency with Nck, with >90% of Nck protein converted to conjugates. Even though the ratio of **p1** to Nck is 5 fold in excess, only one Nck–peptide conjugate band was observed in the SDS-PAGE; the 1:1 reaction ratio suggests that the reaction most likely happens at the peptide-binding groove of Nck-SH2, i.e., with the projected lysine residues. All the peptides carrying sulfonyl fluoride (**p1**, **p5**, **p7**, **p10**, and **p11**) showed noticeable reactivity with Nck, whereas among the chloroacetyl-containing peptides, only **p2** and **p9** showed reactivity. Microscale thermophoresis (MST) analysis also showed that **p1** outperformed **p7** (as one example), giving a significantly lower EC_50_ value, 0.75 vs 6.2 μM respectively (Figure S3 in the Supporting Information). At 37 °C in PBS buffer, the 1:1 reaction efficiency reached 50% in 2 min (Figure S4 in the Supporting Information). These data show that peptides with side-chain-modified sulfonyl fluoride are viable reactive peptides for Nck, and the Nck-peptide binding induces a covalent cross-linking reaction between Nck and the irreversible blocker.

### D Mass Spectrometric Evidence for a Single-Site Lysine Reaction in Nck-SH2

**Figure 5:**
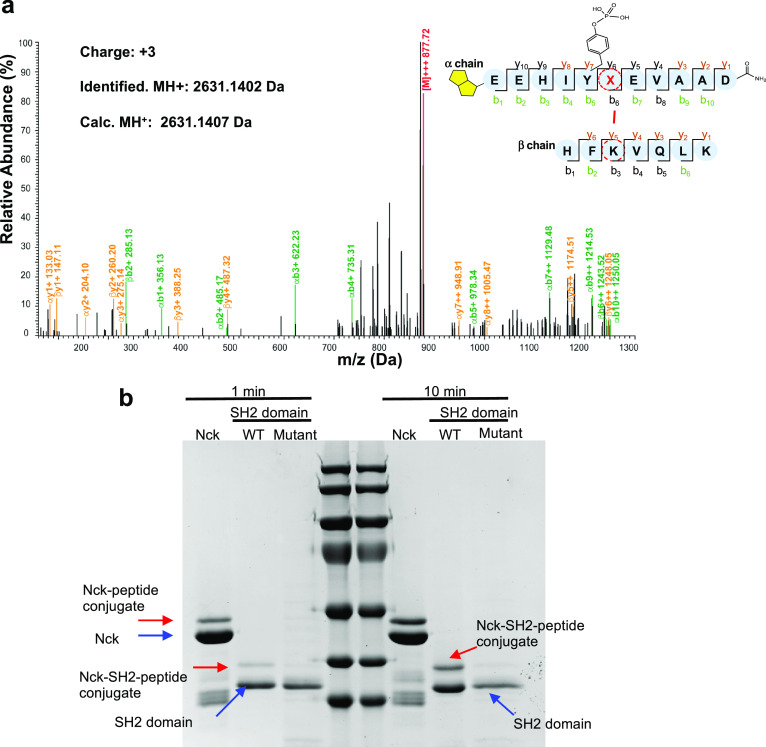
Peptide p1 reacts with Lys 331. (a) MS/MS analysis of the conjugated site between **p1** peptide and Nck-SH2 at Lys331. The corresponding mass data are shown in Figure S6 in the Supporting Information. (b) Mutation of Lys331 to Ala abolished the covalent reaction of Nck with **p1**. Purified recombinant proteins were incubated with peptides in PBS buffer at 37 °C for 1 min (left) or 10 min (right). Molecular weight markers are shown in the middle. Red arrows indicate the covalent peptide–protein complex.

We next identified the residues in Nck-SH2 that are involved in the reaction with **p1** by mass spectrometry. LC-MS analysis first confirmed that only one p1 was conjugated with Nck (Figure S5 in the Supporting Information). The Nck–**p1** conjugate was digested with trypsin, and peptide fragments were analyzed with an in-line EASY-spray source and nano-LC UltiMate 3000 HPLC system interfaced with an Elite mass spectrometer, operated in the data-dependent acquisition (DDA) mode with one full MS scan at *R* = 60,000 (*m*/*z* = 200) mass followed by HCD MS/MS scans. A new peak corresponding to the precursor mass (Mr + 3H)^3+^ of biotinylated **p1** peptide (α-chain) conjugated with Nck-SH2 (β-chain) was observed at 2631.1402 Da after searching by pLink v2.3.11.^37^ The fragmentation spectral analysis of [EEHIpYXEVAAD(α)] + [HFKVQLK(β)] extracted by pLabel v2.4.3.0 (Figure 5a and Figure S6 in the Supporting Information)^38,39^ confirmed the ligand-directed conjugation of Nck-SH2 protein with **p1** peptide. The underlined lysine residue in the β-chain sequence indicated the cross-linked site on the predicted Nck Lys331 residue. Next, we mutated Nck Lys331 to Ala, expressed and purified the Nck-SH2_K331A_ mutant. In contrast to wild-type Nck-SH2 and Nck, Nck-SH2_K331A_ did not show noticeable reactivity with peptide **p1** (Figure 5b). These data identify Lys331 as a reaction site for **p1** on Nck-SH2, suggesting that the proximity-driven lysine reaction occurred in a residue-selective manner.

In addition, we performed the reaction between **p1** and Nck in a cell lysate, representing a complex solution. We incubated peptide **p1** with the lysate of Caco-2 cells overexpressing Nck, and the reaction mixture was resolved on SDS-PAGE and analyzed with Western blotting against an anti-Nck antibody. A complete band shift was observed in the reaction system with **p1**, whereas for other peptides only a partial band shift was observed (Figure S7 in the Supporting Information). Next, we incubated biotinylated peptide **Y474** and Nck solutions with or without **p1** peptide, and pulled down proteins that bound with biotinylated **Y474**. In the absence of **p1**, we clearly observed that Nck bound with **Y474**. However, in the presence of p1, the Nck protein did not bind with **Y474**, suggesting that **p1** competitively blocked the peptide-binding site of Nck-SH2 (Figure S8 in the Supporting Information). These data show that **p1** competitively blocks the protein binding site of Nck-SH2.

### E Nck-p1 Reaction Disrupts LLPS

**Figure 6:**
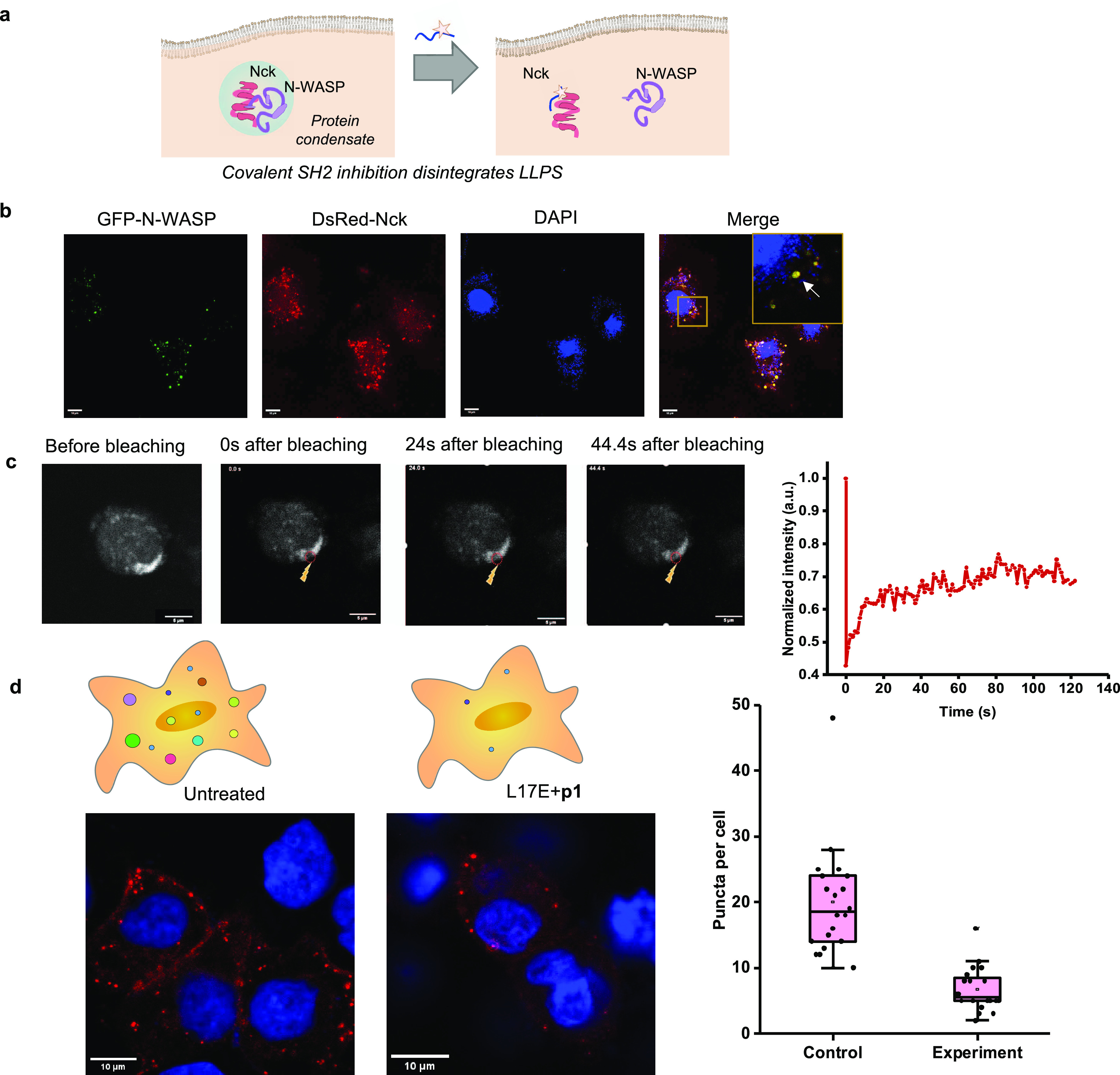
Peptide p1 inhibits the formation of the Nck/N-WASP condensate in mammalian cells. (a) Schematic illustration showing inhibition of intracellular Nck coacervation by **p1**. (b) Confocal microscopic images showing the formation of Nck/N-WASP puncta in HeLa cells that overexpress GFP-N-WASP and dsRed-Nck. Scale bar: 10 μm. (c) FRAP analysis showing the Nck/N-WASP condensates in HeLa cells have fluidity properties based on the recovery of the GFP fluorescent signal after photobleaching in a two-photon microscope with excitation at 920 nm. Scale bar: 5 μm. (d) Confocal microscopic images showing that L17E/**p1**-treatment reduced the numbers of Nck punta in Caco-2 cells transfected to overexpress dsRed–Nck. L17E, 40 μM; **p1**, 1 μM. Statistical analyses of puncta number per cell in the control and experiment (L17E/**p1**). *N* = 20 cells.

To explore whether intracellularly delivered peptide **p1** could disrupt the Nck-mediated phase separation in mammalian cells (Figure 6a), we first examined whether Nck can form coacervates with Nck in mammalian cells. Confocal microscopic images of HeLa cells overexpressing fluorescently tagged Dsred-Nck and GFP-N-WASP revealed the formation of micrometer-sized puncta with colocalized Dsred and GFP signals (Figure 6b). An intracellular FRAP analysis using a home-built two-photon fluorescence microscope at room temperature revealed the fluidlike property of the puncta, showing they are not precipitates (Figure 6c). After photobleaching the GFP fluorescence in puncta to 40% of its original fluorescence intensity, gradual fluorescence recovery to 70% in 50 s was observed. This shows that the Nck/N-WASP condensates are dynamic phase-separated droplets.

Delivering **p1** into mammalian cells across the plasma membrane is a challenge, as the negatively charged **p1** can not spontaneously enter mammalian cells. After exploring several delivery systems, we finally selected the L17E peptide to deliver **p1** into mammalian cells. The lipid-sensitive endosomolytic peptide L17E (IWLTALKFLGKHAAKHEAKQQLSKL-amide) was discovered based on peptide screening and was found to bring cargo into the cytosol through endosomal escape by sampling mixing with the cargo.^40^ Fluorescently labeled **p1** was efficiently delivered into Caco-2 cells by mixing with L17E, and the fluorescence signal was distributed in the cytosol (Figure S9 in the Supporting Information). We also confirmed that L17E-delivered **p1** did not cause noticeable cytotoxicity based on the CCK8 assay (Figure S10 in the Supporting Information). Western blotting using an Nck antibody identified an Nck complex with higher molecular weight in the lysate of L17E/**p1**-treated cells, showing L17E-delivered **p1** reacted with the endogenous Nck in the cytosol of Caco-2 cells (Figure S11 in the Supporting Information). The extent of the molecular weight increase in this case was lower than that *in vitro*, likely due to the intracellular degradation of the **p1** peptide. Despite the fact that **p1** may react with a range of nucleophiles in the cells, here we provide evidence of the reaction between endogenous Nck and **p1**, which paves the way for the following function-based experiments. When Caco-2 cells overexpressing dsRed-Nck were treated with the **p1** peptide (1 μM) and L17E (40 μM) for 2 h, we found a 50% decline in puncta numbers in the cells, showing a significant decrease of phase-separated Nck condensates (Figure 6d). This data show that Nck and N-WASP form phase-separated condensates in cotransfected HeLa cells, and L17E-delivered **p1** peptide may disrupt the Nck/N-WASP coacervates.

### F L17E/p1 Protects Cells against EPEC Infection

**Figure 7:**
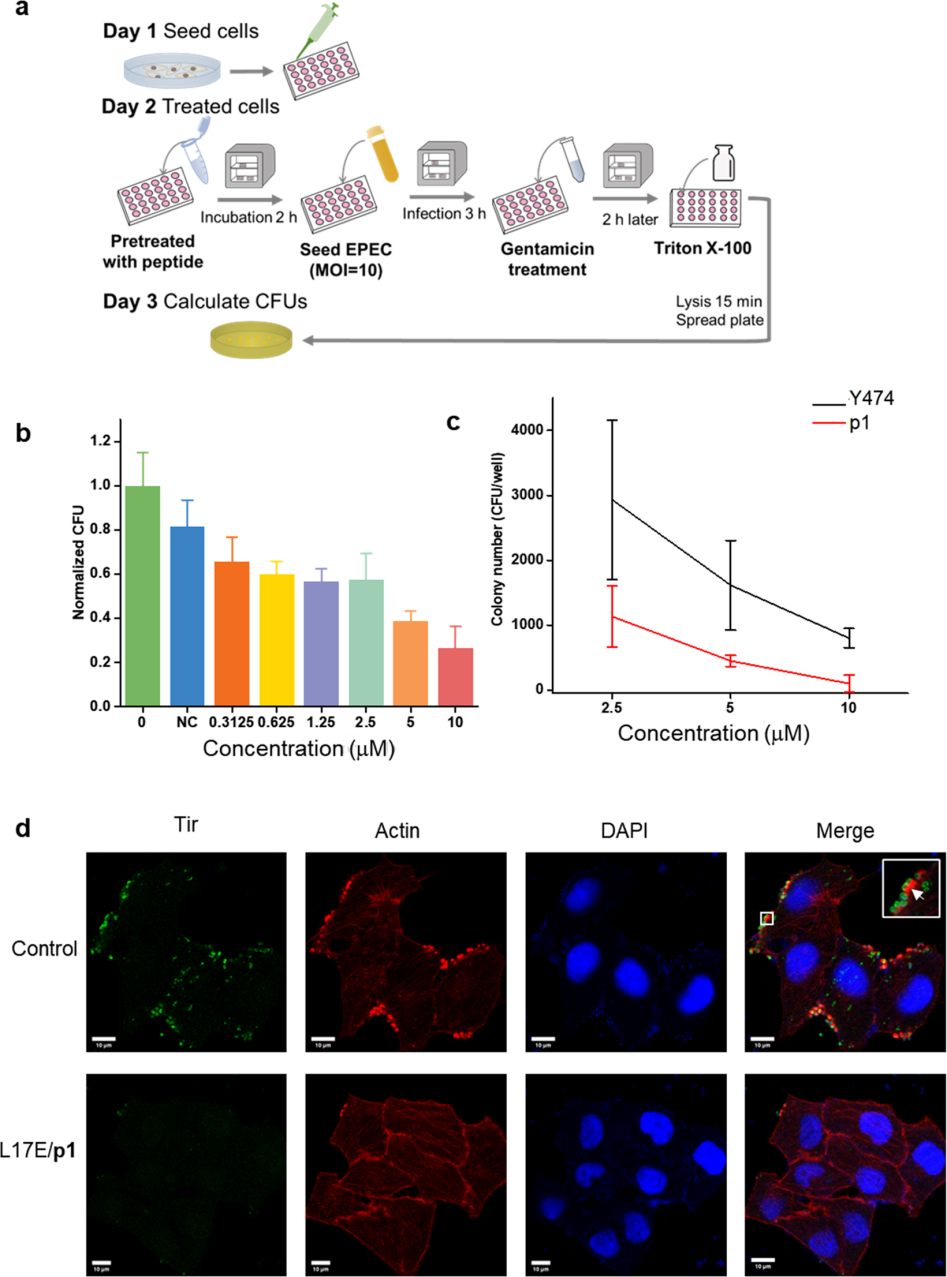
L17E/p1 treatment safeguards Caco-2 cells against EPEC infection. (a) Schematic illustration showing the procedure of the infection assay. (b) Anti-infection efficacy of L17E/p1 treatment at different concentrations of **p1** using CFU as an indicator for infection. (c) Comparison of **p1** and **Y474** peptides in the anti-infection assay. (d) Representative microscopic images of L17E/**p1**-treated Caco-2 cells showing the absence of actin pedestals, whereas in the absence of **p1** peptide, EPEC induces actin pedestal formation to attach to Caco-2 cells. The concentration of L17E in all of the experiments was 40 μM.

Lastly, to examine whether pretreatment of Caco-2 cells with L17E/**p1** could prevent EPEC infection, we first established a bacterial infection assay (Figure 7a). Caco-2 cells were pretreated with L17E/**p1** for 2 h. After excess peptides were removed, Caco-2 cells were then infected with EPEC at different multiplicity of infection (MOI) values (MOI is commonly defined as the ratio of infectious bacteria to cells in a culture) for 3 h, and extra bacterial cells were removed from the culture. Next, gentamicin was added to the cell culture to remove EPEC that are merely attached to the surface of Caco-2 cells, leaving only engulfed EPEC inside Caco2 cells to be counted (gentamicin treatment will not kill intracellular bacteria due to its cellular impermeability). After gentamicin was washed away, cells were lysed to release EPEC cells inside, and the cell lysates were diluted to count the number of EPEC cells as colony-forming units (cfu) on agar plates. L17E/**p1** treatment reduced the CFU numbers in a dose-dependent manner, with 5 μM of **p1** reducing about 50% of the EPEC infection (Figure 7b). Comparing the covalent blocker **p1** and the noncovalent blocker **Y474** peptide, both blockers can disintegrate phase-separated Nck droplets and reduce the turbidity of the solution (Figure S12 and S13 in the Supporting Information). Although both peptides, in conjunction with L17E-mediated delivery, can block EPEC invasion to Caco-2 cells, covalent blocker **p1** showed significantly higher efficacy than **Y474** at lower concentrations (Figure 7c).

Lastly, to confirm that the inhibitors conferred resistance through actin rearrangement and pedestal formation, Tir and actin were visualized in inhibitor-treated cells. Briefly, infected Caco-2 cells were fixed with paraformaldehyde, stained with immunofluorescent anti-Tir antibody, and treated with phalloidin labels actin. EPEC cells were found to be located on the surface of Caco-2 cells and supported by the actin network, showing that EPEC infection induced actin pedestal formation (Figure 7d).^36^ Treatment with L17E/**p1** significantly reduced the number of EPEC cells that attach to the surface of Caco-2: nearly no noticeable EPEC cells can be found under the microscope. Also, actin is evenly distributed on the surface of Caco-2 cells without signs of forming pedestals. These data show that L17E-delivered **p1** prevented the invasion of EPEC into Caco-2 cells by inhibiting Nck-mediated pedestal formation.

## 3 Discussion

Nck bridges the externally introduced EPEC virulence factor Tir with a downstream actin rearrangement signal in an intestine cell and makes the cell a recipient of EPEC. To do so, Nck utilizes its four protein-binding domains, three SH3 and one SH2, to bind with designated proteins upstream and downstream and form a signaling complex. Our work shows that the signaling complex formed between Nck and N-WASP may exist as phase-separated condensates, where phosphorylated nephrin (mimicked by a synthetic peptide **3pY** here) promotes the Nck/N-WASP phase separation and reduces the minimal protein concentrations required for phase separation. Based on this observation, we designed noncovalent and covalently reactive peptides to block the pY-binding site of Nck-SH2. A sulfonyl fluoride-containing pY peptide **p1** covalently reacted with Nck-SH2 at Lys331 and effectively blocked the Nck-mediated phase separation. Through an L17E delivery system, the covalent blocker **p1** reversed the phase separation of Nck and N-WASP and disallowed EPEC from entering Caco-2 cells. Unlike **3pY**, which promoted the LLPS of Nck and N-WASP by providing multivalent interaction sites and flexible internal PEG_4_ linkers, **Y474**-derived monovalent binders at high concentrations competed with the multivalent binders and disrupted the LLPS.

Blockage of a specific SH2 domain is challenging because the binding of pY to the pY-binding pockets on the SH2 domains contributes to about 50% of the binding energy, which makes SH2–peptide binding to be promiscuous. In a previous report, we identified SH2 domains that have special cysteine residues at the peptide-binding site and developed reactive peptides containing an α-chloroacetyl group at selected sites that are adjacent to the cysteine residue. Proximity-driven reactivity enables selective reaction at the cysteine.^30^ Here, we show a proximity-driven lysine reaction at Lys331 of the Nck-SH2 domain. Covalently reactive peptide **p1** showed high reactivity with Nck-SH2 both in the cell lysate *in vitro* and in the cytosol. Admittedly, the sulfonyl fluoride on **p1** also reacts with other nucleophiles inside cells, but these side reactions did not cause noticeable changes in the cell physiology or toxicity. On another note, delivery of the reactive peptide **p1** to the cytosol while retaining its reactivity is not trivial. Covalent linkage of **p1** with positively charged cell-penetrating peptides invalidated the activity of **p1** possibly because of shielding of the pY residue (data not shown). Importantly, the L17E delivery system does not need a covalent linkage with **p1**, and the endosomolytic activity of L17E allows **p1** to escape endosome entrapment and be distributed in the cytosol. However, the drawback of this delivery method is that it is not economical due to the high concentration of L17E peptide required in the delivery: For example, for the delivery of the 1 μM **p1** peptide, 40 μM L17E is required.

Targeting the phase separation property of the signaling protein complex is emerging as a possible way to interfere with cellular signal transduction. Condensate-modifying therapeutics (c-mods), including peptides and small molecules, have been designed to alter condensate behaviors with functional consequences in cell-based studies. For example, Zhou and co-workers identified a peptide targeting the dimerization domain of the SARS-CoV-2 nucleocapsid protein (SARS2-NP) and found it disrupted the SARS2-NP LLPS and inhibited virus replication.^41^ Instead of directly acting on bacterial proteins, the inhibitory blocker in our approach targets an adaptor protein inside the cytosol of the host cells. Blockage of the Nck-SH2 binding site inhibits the Nck LLPS, shuts down Nck-mediated signal transduction, and inhibits bacterial infection. The inhibition of the Nck-SH2 binding site directly results in two events: the disintegration of Nck coacervates and the blockage of EPEC infection. According to Banjade and Rosen, p-nephrin/Nck/N-WASP condensates promote Arp2/3 complex-dependent actin assembly.^28^ Arp2/3-dependent actin polymerization is also known to play a critical role in EPEC infection.^16^ Therefore, disruption of the Nck condensates may affect Arp2/3-dependent actin polymerization and subsequently inhibit EPEC infection. Although direct evidence for the linkage between these events is yet to be acquired, modulating the LLPS of adaptor proteins by blocking essential protein–protein interactions in the host cells may provide a new antibacterial mechanism against the infection of EPEC.

On another note, unlike p-nephrin, the role of phosphorylated Tir (p-Tir) in the Nck LLPS is yet unclear, as neither **3pY** nor peptide **Y474** represents the membrane-bound protein p-Tir. Because peptide **Y474** at low concentrations did not disintegrate the Nck/N-WASP/**3pY** coacervates, it is possible that the membrane-bound p-Tir (with the C-terminal Y474 phosphorylated) at low concentrations may join the Nck/N-WASP/p-nephrin coacervates. Banjade and Rosen’s work suggests that intimin on the surface of EPEC may cluster p-Tir and promote the formation of Nck coacervates,^28^ which consequently leads to pedestal formation during the EPEC infection. The role of p-Tir clearly needs more investigation.

## Supporting Information Available

Methods and experimental procedures; colocalization of Cy3-N-WASP and Cy5-Nck in Nck/N-WASP/3pY microdroplets; microscale thermophoresis (MST) experiment to measure the binding of Y474 to Nck; MST experiment measured the EC50 of peptide 1 and 7; reaction kinetics; LC-MS analysis of Nck and Nck-p1 conjugate; mass data of the selected peaks in Figure 5a; reaction of biotin-p1 to cell lysate; a competitive pull-down experiment to show Nck-p1 complex failed to bind to peptide Y474; L17E delivers p1 peptide into the cytosol of Caco-2 cells; cell viability analysis by CCK8 assay; in cell reaction; peptide p1 reverses the phase separation of Nck/N-WASP/3pY; peptide Y474 reverses the phase separation of Nck/N-WASP/3pY; chemical structure of all the peptides and the mass spectrum data (PDF)
