# Supplementary material for: Modulating Liquid–Liquid Phase Separation of Nck Adaptor Protein against Enteropathogenic Escherichia coli Infection
Source: ACS Cent Sci. 2023 Dec 14;9(12):2358–68. doi: 10.1021/acscentsci.3c01068 (PMC10755736; doi:10.1021/acscentsci.3c01068)
Supplement: Supplementary file 1 — oc3c01068_si_001.pdf [file oc3c01068_si_001.pdf]

Supporting Information

Modulating Liquid-Liquid Phase Separation of Nck Adaptor Protein against Enteropathogenic *Escherichia coli* Infection

Min Liu,†,[a] Chunjian Wu,†,[a,*] Rui Wang,[b] Jiaming Qiu,[a] Zhentao She,[c] Jianan Qu,[c] Jiang Xia*,[a]

[a] Department of Chemistry and Center for Cell & Developmental Biology, The Chinese University of Hong Kong, Shatin, Hong Kong SAR, China.

[b] Pingshan Translational Medicine Center, Shenzhen Bay Laboratory, Shenzhen, 518118, China.

[c] Departments of Electronic and Computer Engineering, Center of Systems Biology and Human Health, School of Science and Institute for Advanced Study, Hong Kong University of Science and Technology, Clear Water Bay, Kowloon, Hong Kong SAR, China.

*Address correspondence to ericdotwug@gmail.com and jiangxia@cuhk.edu.hk ORCID 0000-0001-8112-7625

Phone: (852) 3943 6165

Fax: (852) 2603 5057

Contents

Items	Pages
**Methods and Experimental Procedures**	S4
**Table S1**. Plasmids used in this study.	S10
**Table S2.** Sequences of proteins used in this study.	S11
**Figure S1.** Co-localization of Cy3-N-WASP and Cy5-Nck in Nck/N-WASP/**3pY** microdroplets.	S13
**Figure S2.** Microscale thermophoresis (MST) experiment to measure the binding of **Y474** to Nck.	S14
**Figure S3.** MST experiments measuring the Nck binding of **p1** and **p7**.	S15
**Figure S4.** Reaction kinetics.	S16
**Figure S5.** LC-MS analysis of Nck and Nck-**p1** conjugate.	S17
**Figure S6.** Mass data of the selected peaks in Figure 5a.	S18
**Figure S7.** Reaction of **p1** in cell lysate.	S19
**Figure S8.** A competitive pull-down experiment to show Nck-**p1** complex failed to bind to peptide **Y474**.	S20
**Figure S9.** L17E delivers **p1** peptide into the cytosol of Caco-2 cells.	S21
**Figure S10.** Cell viability analysis by CCK8 assay.	S22
**Figure S11.** In cell reaction.	S23
**Figure S12.** Peptide **p1** reverses the phase separation of Nck/N-WASP/3pY.	S24
**Figure S13.** Peptide **Y474** reverses the phase separation of Nck/N-WASP/3pY.	S25
**Figure S14.** Chemical structure of 3pY and the ESI mass spectrum data.	S26

**Figure S15.** Chemical structure of 3Y and the ESI mass spectrum data.	S27
**Figure S16.** Chemical structure of p1 and the mass spectrum data by MALDI-TOF.	S28
**Figure S17.** Chemical structure of p2 and the mass spectrum data by MALDI-TOF.	S29
**Figure S18.** Chemical structure of p3 and the mass spectrum data by MALDI-TOF.	S30
**Figure S19.** Chemical structure of p4 and the mass spectrum data by MALDI-TOF.	S31
**Figure S20.** Chemical structure of p5 and the mass spectrum data by MALDI-TOF.	S32
**Figure S21.** Chemical structure of p6 and the mass spectrum data by MALDI-TOF.	S33
**Figure S22.** Chemical structure of p7 and the mass spectrum data by MALDI-TOF.	S34
**Figure S23.** Chemical structure of p8 and the mass spectrum data by MALDI-TOF.	S35
**Figure S24.** Chemical structure of p9 and the mass spectrum data by MALDI-TOF.	S36
**References**	S37

Method and Experimental Procedures

Materials and Instruments. Unless otherwise specified, all reagents and solvents were purchased from commercial sources and were used without further purification. Cytotoxicity assay kits were purchased from MedChemExpress (USA). Dulbecco's modified Eagle medium (DMEM), fetal bovine serum (FBS), phosphate-buffered saline (PBS), and Penicillin-Streptomycin-Glutamine (100X) liquid were purchased from Gibco™ (Thermo Fisher Scientific, USA).

Protein Expression and Purification

All plasmids used in this work are listed in Table S1. The sequences of proteins are listed in Table S2. Recombinant proteins were expressed in BL21(DE3) cells at 18 °C for overnight after induction. Cells were centrifuged and lysed by sonication in the lysis buffer. For Nck, Nck-SH2, and Nck-SH2 $_{K331A}$, the lysis buffer used was 20 mM Tris, 500 mM NaCl, 3 mM DTT, and the elution buffer was 20 mM Tris, 250 mM NaCl, 3 mM DTT, and 10 mM reduced glutathione. Cell lysates were centrifuged to remove the debris, applied to the GSH agarose resin (Cytiva, Hong Kong), washed with the lysis buffer, and eluted with the elution buffer. To remove the GST tag of Nck protein, Nck was treated with HRV 3C protease (Thermo Fisher Scientific, USA) at 4 °C for overnight. Furthermore, the protein was separated by a Superdex 75 10/300 GL column (Cytiva, Hong Kong) in 25 mM HEPES, pH 7.5, 150 mM NaCl, and 2 mM beta-mercaptoethanol (βME). For N-WASP, the lysis buffer contained 20 mM imidazole, 300 mM KCl, 5 mM βME, 0.01% NP-40, 1 mM PMSF, and 1 mM benzamidine at pH 7. The wash buffer was 300 mM KCl, 50 mM imidazole, pH 7, and 5 mM βME. And the elution buffer was 100 mM KCl, 300 mM imidazole, pH 7, and 5 mM βME. The lysate was centrifuged to remove the debris, applied to Ni-NTA agarose (Thermo Fisher Scientific, USA), washed with the wash buffer and eluted with the elution buffer. To remove the His tag, N-WASP was treated with TEV protease (Thermo Fisher) at 4 °C for overnight. Furthermore, the protein was separated by a Source 15 S column

(Cytiva, Hong Kong) using a gradient from 100 to 500 mM NaCl and Superdex 75 10/300 GL column (Cytiva, Hong Kong) in 25 mM HEPES, 150 mM NaCl, and 2 mM βME at pH 7.5.

Protein Labeling

Proteins were buffer-exchanged through a Hi-trap desalting column (Cytiva, Hong Kong) to the reaction buffer. For Cy5 labeling, the buffer was 0.1 M sodium bicarbonate buffer at pH 8.3. For FITC labeling, the buffer was 50 mM sodium borate at pH 8.5. At a 1:1 molar ratio, the protein was mixed with Sulfo-Cy5 NHS ester or FITC and incubated for 2 h. To remove the excessive small molecules, the products were buffer-exchanged through a Hi-trap desalting column (Cytiva, Hong Kong) to PBS buffer. Purified proteins were stored at -80 °C for further analysis.

Turbidity Assay

Proteins and peptides were mixed to form protein condensates under different conditions, and the optical density at 600 nm was observed in 384 well plates using the microplate reader spectrophotometer (Thermo Fisher Scientific, USA).

Fluorescence Recovery after Photobleaching (FRAP) Assay

Fluorescence recovery *in vitro* was conducted with a DMi8 microscope (Leica, Germany). Protein condensate was observed on a confocal dish under 100 × oil immersion objective. The samples were photobleached by irradiation with laser at the droplet for 2 s, and the images were captured for 5 min. The fluorescence intensity was quantified and normalized by ImageJ.

Fluorescence recovery after photobleaching experiments inside mammalian cells was conducted with a two-photon imaging system using a custom-built upright microscope, based on a previously used imaging setup. [1] Briefly, the excitation light was delivered by a tuneable mode-locked titanium:

sapphire laser (Chameleon Ultra II, Coherent, USA). After collimation by a variable beam expander, the laser beam was directed to X-Y galvanometric scan mirrors (6210H, Cambridge Technology, USA). The intermediate plane of the Galvo-X and Galvo-Y mirrors was conjugated to the back focal plane of a water-immersion objective (XLUMPLFLN20XW, 1.0 NA, 2 mm W.D., Olympus, Tokyo, Japan) *via* a scan (LSM03- BB, Thorlabs, NJ, USA) and a tube lens (TTL200MP, Thorlabs). The fluorescence collected by the objective was separated from the excitation light by a dichroic mirror (705LP, Chroma ATE). Green fluorescence protein was excited at a wavelength of 920 nm, and the fluorescence was directed to one photomultiplier (PMT) module (H7422, Hamamatsu, Japan). One band-pass filter (FF03-525/50-25, Semrock, USA) placed before the PMTs was used to select the fluorescence wavelength for detection. The microscope was controlled, and images were acquired using a multifunction data-acquisition (DAQ) device (PCIe-6361, National Instruments, USA) running a customer-written C# program.

Peptide Synthesis and Characterization

All the peptides were synthesized using standard Fmoc-based peptide synthesis chemistry. Rink Amide-ChemMatrix resins with a loading capacity of 0.4-0.5 mmol/g were purchased from Biotage (Uppsala, Sweden). Fmoc amino acids were coupled in five-fold excess with HBTU, HOBT, and DIEA (1:1:2) in DMF for 30 min. Fmoc deprotection was performed by treating the peptide-bound resin with 20% (v/v) piperidine/DMF for 20 min. After that, the resins were thoroughly washed with DMF and DCM. After assembly of the peptide chain, the N-terminus of the peptide was labeled with 5, or 6-Carboxyfluorescein (FAM) or biotin. After assembly, the Alloc group was deprotected using Pd(PPh3)3/PhSiH3 in DCM, and subsequently, 3-(fluorosulfonyl)benzoic acid was coupled to the amino side chain of diaminopropionic acid (Dap) using five-fold excess of HBTU, HOBT, and DIEA (1:1:2) in DMF for 1 h. The final deprotection and cleavage of the peptides from the resin was realized using TFA/H_2O/TIS (95/2.5/2.5, v/v). Crude peptides were precipitated by the addition of cold diethyl

ether and purified by reverse-phase HPLC on a C18 column using acetonitrile/H_2O (with 0.1% TFA) gradient on a reversed-phase C18 column, confirmed by MALDI-TOF MS analysis (Bruker, USA). Pure peptides were lyophilized.

Crosslinking of Peptides with Nck *in vitro*

Purified peptides were incubated with the recombinantly expressed Nck protein (10 μM) in PBS buffer at 37 °C. After incubation, 5× loading buffer was added into the solution and heated at 100 °C for 10 min. These samples were then separated by 12% SDS-PAGE gel, which contained 10% glycerol instead of DI water, followed by staining with Coomassie brilliant blue. Western blot was then performed using the primary antibody specific for Nck or biotin and the secondary antibody.

Mass Spectrometric Analysis

The reaction mixture of Nck and **p1** were digested with trypsin. Digested peptides were analyzed with an in-line EASY-spray source and nano-LC UltiMate 3000 high-performance liquid chromatography system (Thermo Fisher, USA) interfaced with an Elite mass spectrometer (Thermo Fisher, USA). The eluted peptides were detected by the Elite mass spectrometer, which was operated in data-dependent mode with one full MS scan at R = 60,000 (m/z = 200) mass, followed by MS/MS scans. Mass spectrometry raw data was searched by Maxquant.

Protein–Peptide Conjugation Reactions and the Binding Assay

The reaction solutions were then thermally denatured in the presence of loading dye to stop the reactions. After being resolved by SDS/PAGE, the gel was first observed under a fluorescent microscope and then stained with Coomassie brilliant blue dye. For the protein-peptide binding assay, recombinantly expressed Nck protein (2 mg) was coated in 96-well plates in carbonate/bicarbonate buffer (pH 9.6). After washing and blocking by nonfat dry milk, biotin-labeled peptides at different

concentrations were added to each well, followed by a 90-min incubation at 37 °C. Diluted streptavidin-HRP solution was then added, followed by substrate solution to develop cover at 450 nm, and recorded by a plate reader. For MST test, a series diluted solutions of the cy5 labeled peptides were incubated with 400 nM Nck for 30 min and loaded into 16 capillaries. And then the samples were scanned and measured at 20% IR Laser power with 30 s laser-on time on a Monolith NT.115 instrument (NanoTemper Technologies). The data were analyzed by K_D fitting model (K_D) or hill fitting model (EC_{50}).

A Cell Line Stably Expressing N-WASP

To establish a stable pool of cells for N-WASP overexpression, Caco-2 cells were infected at 70-80% confluency with the virus diluted in a suitable medium supplemented with 8 g/ml polyene. After 48 h of incubation, infected cells were treated with puromycin for 7-14 days. The selection medium was changed every 2 days. Under the same treatment conditions, uninfected parental cells were used as selection controls. All antibiotic-resistant cells were pooled together as stable populations and maintained in the presence of low concentrations of puromycin (1-8 μg/ml) for further analysis. Overexpression N-WASP was confirmed by real-time PCR and immunoblotting analysis.

Cell Transfection and Viability

Plasmid extraction was done using a plasmid DNA Maxi kit (Omega Bio-Tek, GA, USA). HeLa or Caco-2 cells stably transfected with pGFP-Nwasp were transfected with pDsred-Nck by Lipofectamine 2000 (Invitrogen, MA, USA) in accordance with the manufacturer's instructions. 48 h after transfection, cells were analyzed by fluorescent micrscopes. Cell viability was measured by a CCK8 assay (Thermo Fisher Scientific) according to its manual.

Bacterial Counts

Caco-2 cells were seeded in a 24-well plate at a density of 1×10^4 cells for 24 h and pretreated with peptide complexes for 2 h. After washing with DMEM, cells were infected with EPEC at selected MOI values for 3 h at 37 °C. Cells were then treated with gentamicin (100 μg/μL) for 2 h and lysed with 1% Triton X-100 for 15 min. The lysates were diluted 100 or 1000 folds to be spread on LB agar plates for colony counting.

Immunofluorescence Confocal Microscopy

Infected cells were fixed with 4% PFA and treated with 0.1% Triton X-100 for 10 min. And then the samples were blocked with 3% BSA for 1 h, incubated with anti-Tir antibody (Abbexa, Cambridge, UK) at 1:200 at 4°C overnight and followed by incubation with Alexa Fluo 488 goat anti-rabbit IgG secondary antibody at room temperature for 2 h. Cells were treated with DAPI (Invitrogen) for 10 min, then stained with phalloidin (Alexa Fluor 594-conjugated phalloidin, Invitrogen) for 1 h and imaged by a confocal fluorescent microscope (SP8, Leica).

Western Blot Analysis

Caco-2 cells were treated with peptides overnight and lysed via RIPA buffer containing protease inhibitors for 15 min at 4°C. The sample was loaded onto 12% SDS/PAGE glycerol gel and then transferred onto a PVDF membrane using Bio-Rad MicroPulser Electroporator. The membrane was blocked and incubated with a rabbit anti-Nck antibody (Abcam, Hong Kong) at 4 °C overnight, followed by an anti-rabbit HRP-labeled secondary antibody (Abcam, Hong Kong).

Statistical Analysis

SPSS 23.0 statistical software was used to process experimental data. The differences between mean ± SEM of three independent experiments were assessed by Student's t-test.

Table S1. Plasmids used in this study.

**Plasmids**	**Backbone**	**Description**	**Source**
pGEX-6P-1-Nck	pGEX-6P-1	N-terminal GST-tagged Nck, Amp^+	[2]
pN-WASP-BPVCA	pET's His-Tev	N-terminal His-tagged N-WASP, Amp^+	[3]
pET21a-Nck-SH_2	pET21a(+)	C-terminal His-tagged Nck-SH_2 , Amp^+	This study
pET21a-Nck-SH_2-K50	pET21a(+)	C-terminal His-tagged Nck-SH_2-K50, Amp^+	This study
pDsRed-Nck	pDsRed-Express-N1	C-terminal Dsred-fusion Nck, Kan^+	This study

Table S2. Sequences of proteins used in this study.

Sequence of Nck:

MSPILGYWKIKGLVQPTRLLLEYLEEKYEEHLYERDEGDKWRNKKFELGLEFPNLPYYIDGD

VKLTQSMAIIRYIADKHNMLGGCPKERAEISMLEGAVLDIRYGVSRIAYSKDFETLKVDFLSK

LPEMLKMFEDRLCHKTYLNGDHVTHPDFMLYDALDVVLYMDPMCLDAFPKLVCFKKRIEA

IPQIDKYLKSSKYIAWPLQGWQATFGGGDHPPKSDLEVLFQGPLGSMAEEVVVVAKFDYVA

QQEQELDIKKNERLWLLDDSKSWWRVRNSMNKTGFVPSNYVERKNSARKASIVKNLKDTL

GIGKVKRKPSVPDSASPADDSFVDPGERLYDLNMPAYVKFNYMAEREDELSLIKGTKVIVME

KCSDGWWRGSYNGQVGWFPSNYVTEEGDSPLGDHVGSLSEKLAAVVNNLNTGQVLHVVQ

ALYPFSSSNDEELNFEKGDVMDVIEKPENDPEWWKCRKINGMVGLVPKNYVTVMQNNPLT

SGLEPSPPQCDYIRPSLTGKFAGNPWYYGKVTRHQAEMALNERGHEGDFLIRDSESSPNDFS

VSLKAQGKNKHFKVQLKETVYCIGQRKFSTMEELVEHYKKAPIFTSEQGEKLYLVKHLS*

(Blue: Precission enzyme site; Red: Nck protein) *

Sequence of N-WASP:

GSEFKEKKKGKAKKKRAPPPPPPSRGGPPPPPPPPHSSGPPPPPARGRGAPPPPPSRAPTAAPPP

PPPSRPGVVVPPPPPNRMYPHPPPALPSSAPSGPPPPPPLSMAGSTAPPPPPPPPPPPGPPPPPGL

PSDGDHQVPASSGNKAALLDQIREGAQLKKVEQNSRPVSCSGRDALLDQIRQGIQLKSVSDG

QESTPPTPAPTSGIVGALMEVMQKRSKAIHSSDEDEDDDDEEDFEDDDEWED*

Sequence of Nck-SH$_2$ domain:

MWYYGKVTRHQAEMALNERGHEGDFLIRDSESSPNDFSVSLKAQGKNKHFKVQLKETVYC

IGQRKFSTMEELVEHYKKAPIFTSEQGEKLYLVKHLSLE*

Sequence of Nck-SH$_2$-K50 domain:

MWYYGKVTRHQAEMALNERGHEGDFLIRDSESSPNDFSVSLKAQGKNKHFAVQLKETVYC

IGQRKFSTMEELVEHYKKAPIFTSEQGEKLYLVKHLSLE*

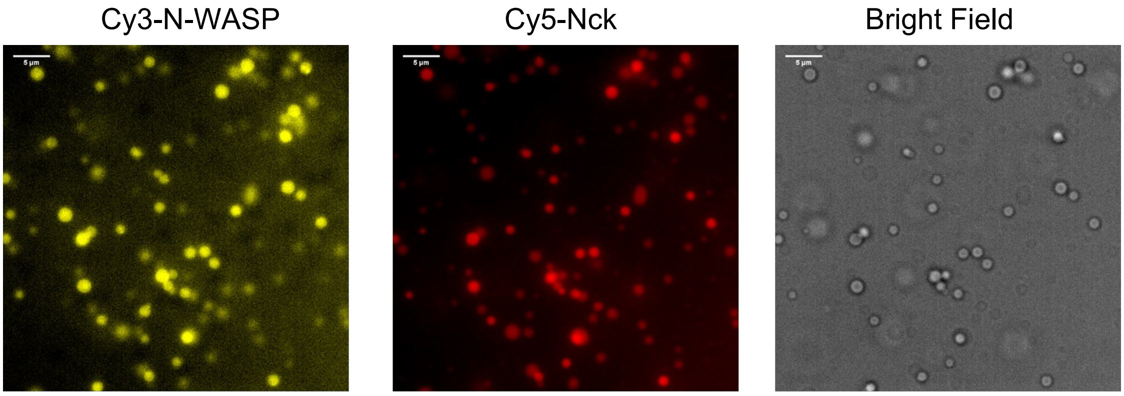

Figure S1. Co-localization of Cy3-N-WASP and Cy5-Nck in Nck/N-WASP/3pY microdroplets.

Nck:15 µM, N-WASP: 5 µM, **3pY**:5 µM. Scale bar: 2 µm.

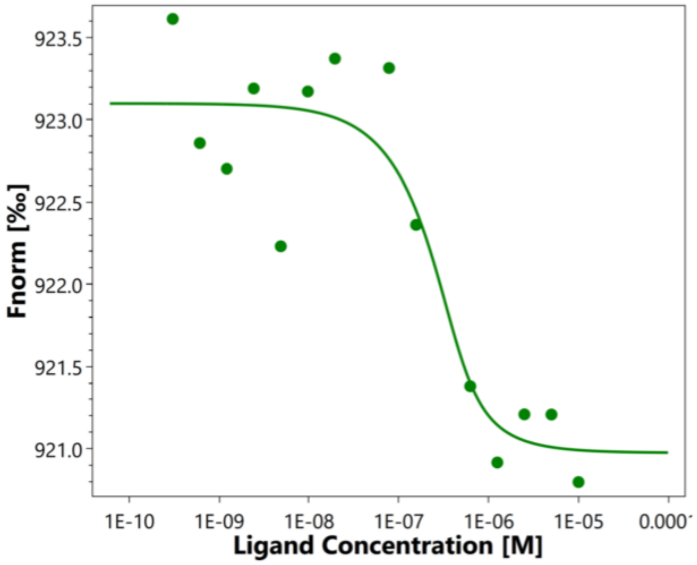

Figure S2. Microscale thermophoresis (MST) experiment to measure the binding of Y474 to Nck. A dissociation constant (K_D) of 76.4 nM was derived from the binding curve. The concentration of Nck was set at 400 nM, and the peptide was titrated into the solution up to 10 μM.

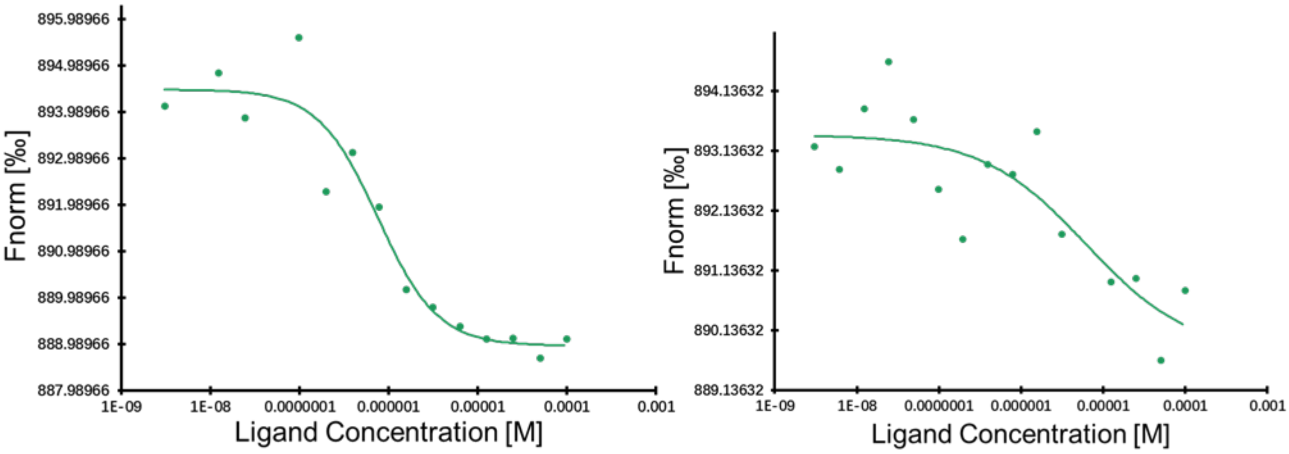

Figure S3. MST experiments measuring the Nck binding of p1 and p7. Left: **p1** (EC_{50}: 0.75±0.24 μM). Right: **p7** (EC_{50}: 6.19±1.34 μM). EC50 was derived from the binding curve from hill fitting model.

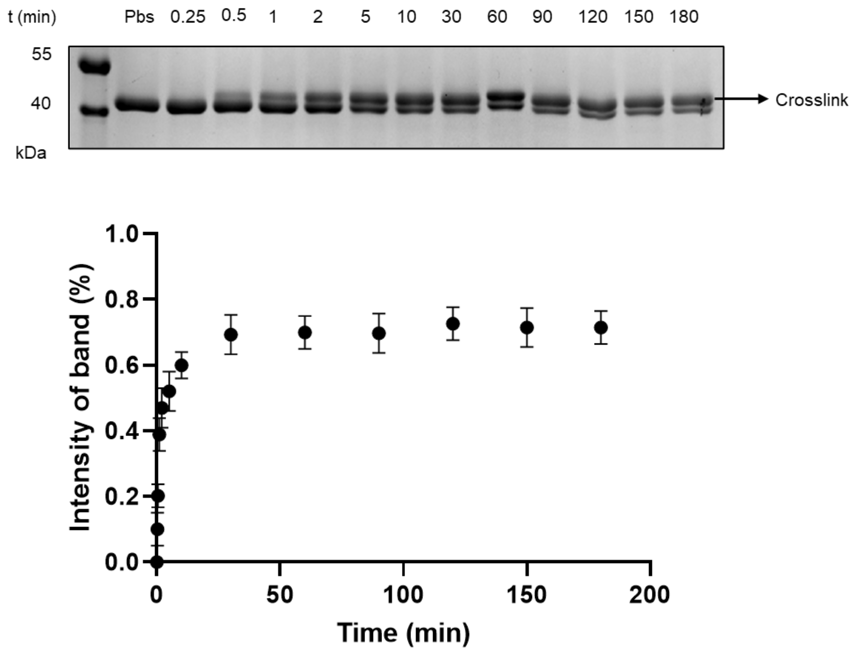

Figure S4. Reaction kinetics. SDS-PAGE analysis of **p1** (5 µM) crosslinking with Nck (5 µM) in PBS buffer at 37 ^{o}C at indicated time points. Black arrows indicate the covalent complex of Nck/p1.

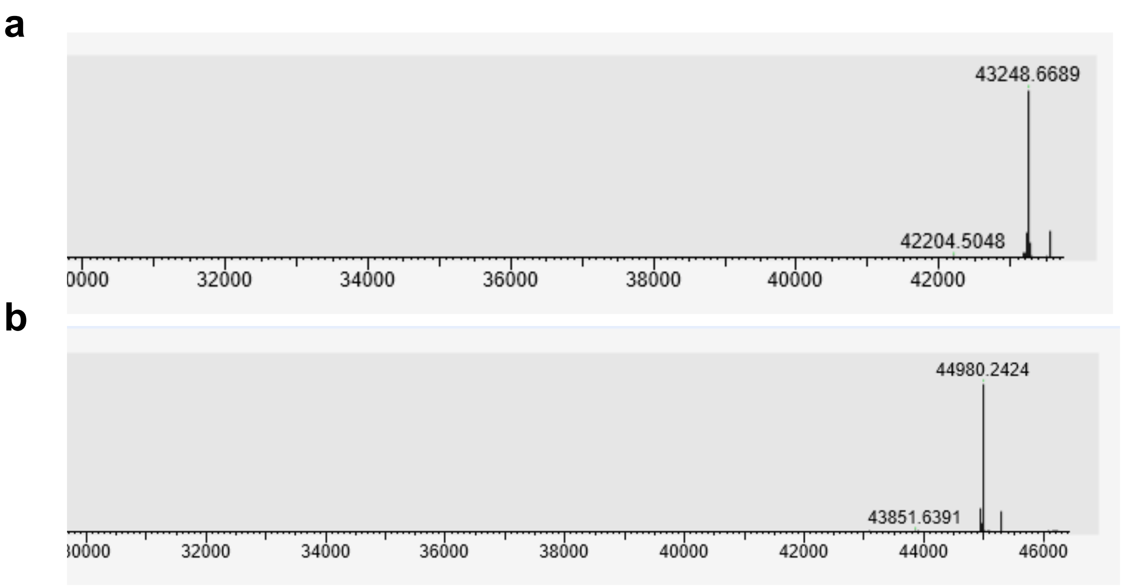

Figure S5. LC-MS analysis of Nck and Nck-p1 conjugate. (a) Recombinant Nck protein shows a molecular weight of 43248.6689 on MALDI-TOF. Expected molecular weight: 43275.92. (b) The Nck–**p1** complex shows a molecular weight of 44980.2424 with a difference of 1731.5735, close to the molecular weight of **p1** with HF removed, 1731.5909.

α-chain		b^+	b++	y+	y++	
E-Biotin	1	356.12747	178.56737			
E	2	485.17006	243.08867	2276.02054	1138.51391	10
H	3	622.22897	311.61812	2146.97795	1073.99261	9
I	4	735.31304	368.16016	2009.91904	1005.46316	8
Y-Phospho	5	978.34270	489.67499	1896.83498	948.92113	7
X	6	2128.90201	1064.95464	1653.80532	827.40630	6
E	7	2257.94460	1129.47594	503.24600	252.12664	5
V	8	2357.01302	1179.01015	374.20341	187.60534	4
A	9	2428.05013	1214.52870	275.13500	138.07114	3
A	10	2499.08724	1250.04726	204.09788	102.55258	2
D-Amidated				133.06077	67.03402	1

β-chain		b^+	b++	y+	y++	
H	1	138.06623	69.03312			
F	2	285.13465	142.56733	2496.09728	1248.04864	6
K	3	2146.83961	1073.41981	2349.02887	1174.51444	5
V	4	2245.90802	1122.95500	487.32390	243.66195	4
Q	5	2373.96600	1186.98500	388.25549	194.12775	3
L	6	2487.05066	1243.52500	260.19691	130.09846	2
K				147.11285	73.55643	1

Figure S6. Mass data of the selected peaks in Figure 5a. The specified b/y ions of the labeled peak in the fragment spectrum of **p1** peptide (a-chain) crosslinking with Nck-SH2 (b-chain). The set of fragment ion types displayed with different colors according to the highlighting matched peaks in MS/MS spectrum. The numbers are color-coded and match the color of peaks in Figure 5a.

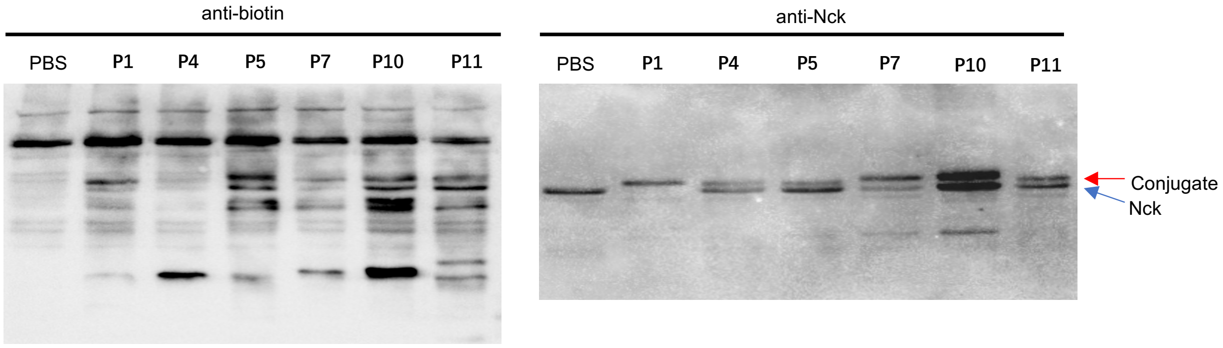

Figure S7. Reaction of p1 in cell lysate. Biotinylated **p1** peptide (10 μM) was incubated with the lysate of Caco-2 cells (5 μg/μl) to react at 37 °C for 60 min before the mixture was resolved on SDS-PAGE. Western blotting experiments using an anti-biotin antibody and an anti-Nck antibody, respectively.

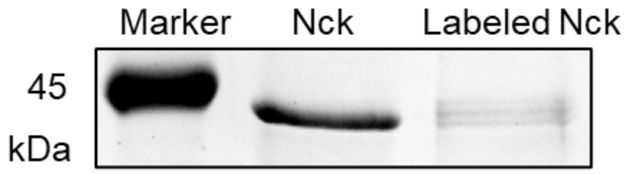

Figure S8. A competitive pull-down experiment to show Nck-p1 complex failed to bind to peptide Y474. Streptavidin MagBeads were incubated with biotinylated **Y474** peptide at 4 °C with continuous rotation for 10 h. Nck proteins (10 μM) unreacted or reacted with **p1** peptide were added to bind with the biotinylated **Y474** peptide modified beads at 4 °C for 5 h. The bound proteins were washed from the beads and analyzed by SDS/PAGE.

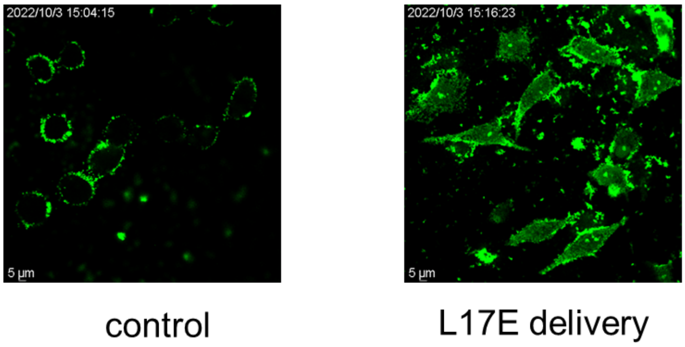

Figure S9. L17E delivers p1 peptide into the cytosol of Caco-2 cells. Left: p1. Right: L17E/p1. 80-90% confluence cells were treated with a mixture of 10 μM FAM-labeled **p1** peptide and L17E peptide (40 μM) in α-MEM(–) for a duration of 3 h at 37 °C. And then the cells were rinsed with α-MEM(+) and recovered with serum-supplemented α-MEM (α-MEM(+)) for another 3 h at 37 °C. The process of cellular uptake of **p1** peptide was then observed in live cells via a Leica confocal laser scanning microscope.

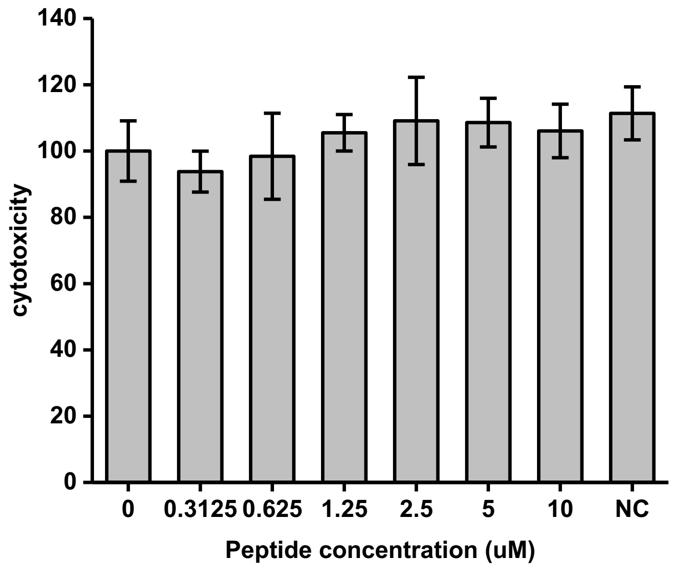

Figure S10. Cell viability analysis by CCK8 assay. Cells were prepared for 8000 cells per well in a 96-well plate, pre-cultured overnight and then treated with **p1** peptide at different concentrations, along with L17E peptide (40 μM) for 3 h. After washed, the cells were added 10 μL of CCK-8 solution and incubated for 48 h. The viability was measured at 450 nm absorbance.

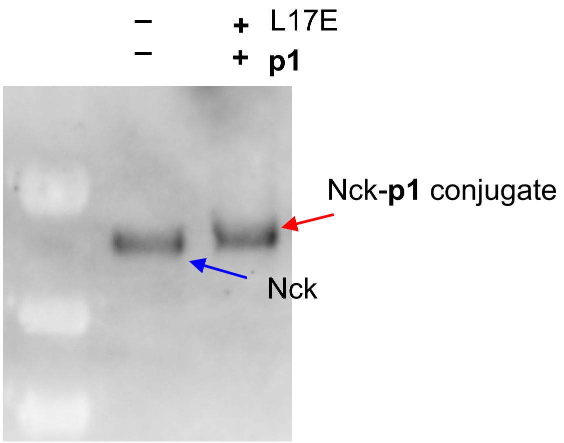

Figure S11. In cell reaction. Covalent reaction of **p1** with Nck protein in Caco-2 cells probed by Western blot analysis using an anti-Nck antibody. A mixture of L17E and **p1** (40 mM and 10 mM, respectively) was incubated with Caco-2 cells overnight before cell lysate and analysis.

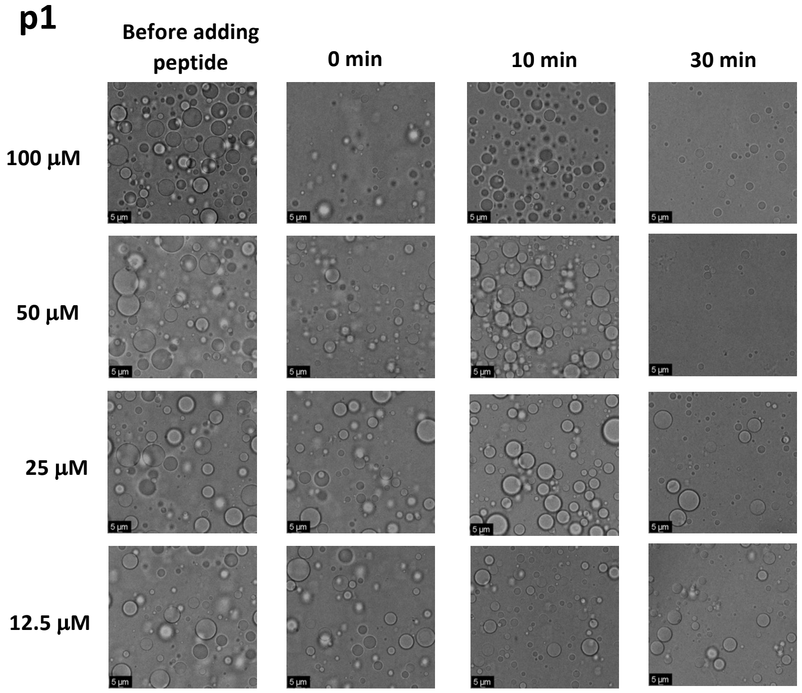

Figure S12. Peptide **p1** reverses the phase separation of Nck/N-WASP/3pY.

Peptides p1 with different concentrations were added to decrease the droplet formation of the Nck/N-WASP/3pY solution and droplet disassembles were observed under confocal microscopy at different times (0 min, 10 min and 30 min). Nck: 80 µM, N-WASP: 15 µM, 3pY: 10 µM.

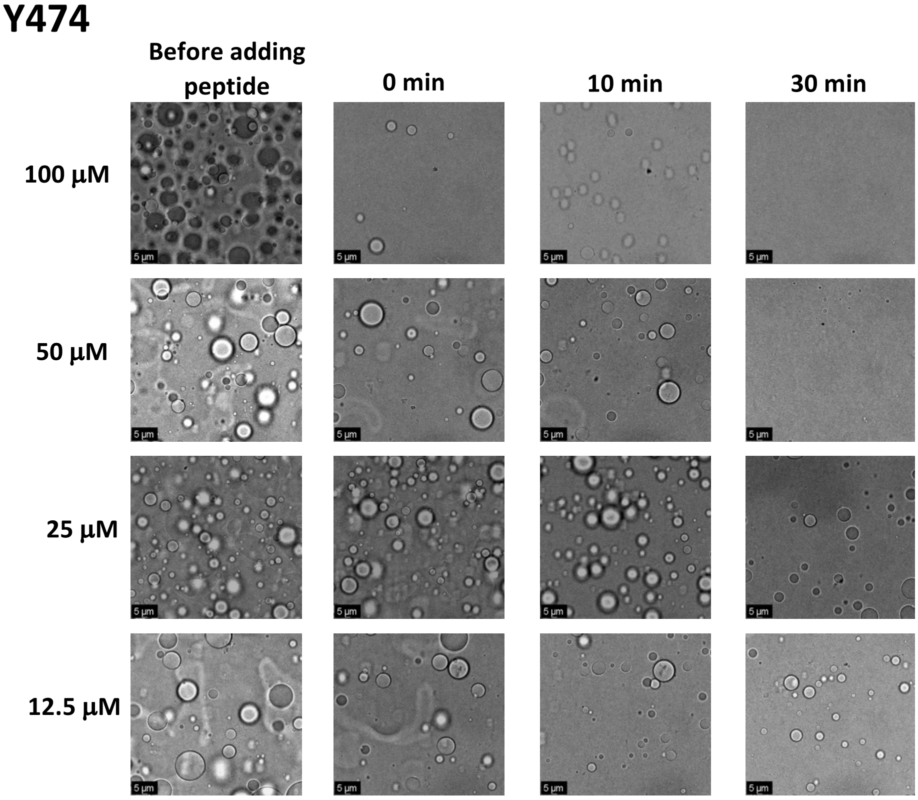

Figure S13. Peptide **Y474** reverses the phase separation of Nck/N-WASP/3pY. Peptides Y474 with different concentrations were added to decrease the droplet formation of the Nck/N-WASP/3pY solution and droplet disassembles were observed under confocal microscopy at different times (0 min, 10 min and 30 min). Nck: 80 μM, N-WASP: 15 μM, 3pY: 10 μM.

The structures and characterization data of synthetic peptides are shown in **Figures S14** to **S24** below.

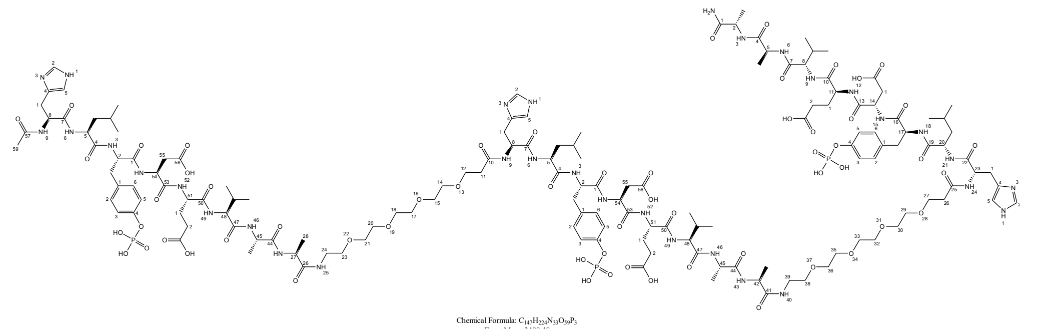

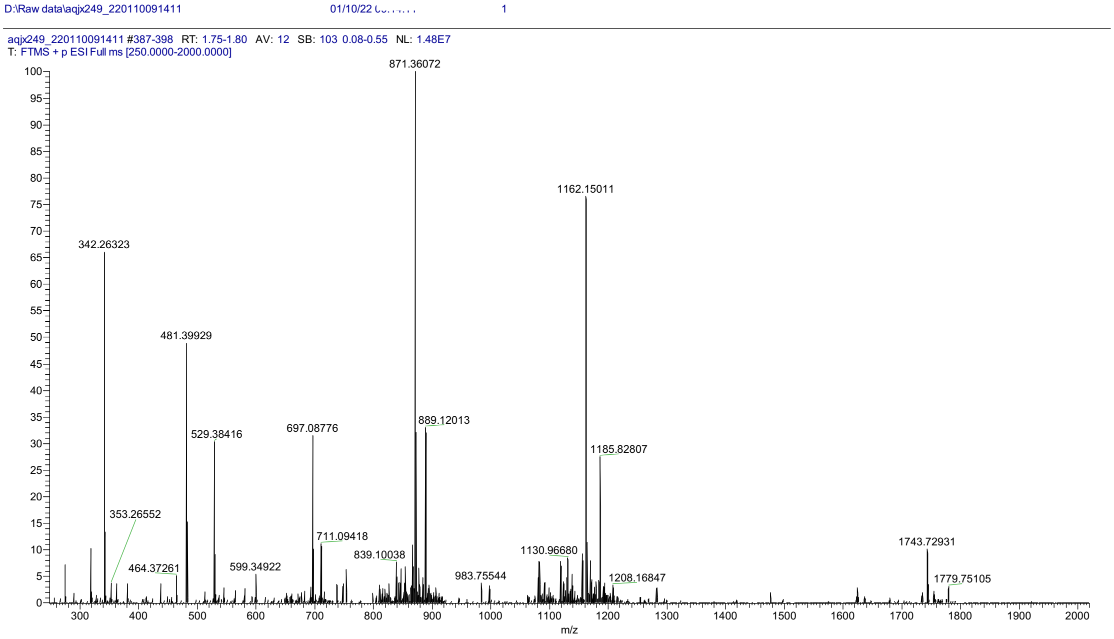

Molecular formula :	$C_{147}H_{224}N_{33}O_{59}P_3$
Experimental Mass $[M\text{-}2H]^{2-}$: $[M\text{-}3H]^{3-}$, $[M\text{-}4H]^{4-}$:	1743.72931, 1162.15011, 871.36072
Theoretical Mass $[M\text{-}2H]^{2-}$: $[M\text{-}3H]^{3-}$, $[M\text{-}4H]^{4-}$:	1743.73196, 1162.15221, 871.36234
Error (ppm) :	-1.5, -1.8, -1.9

Figure S14. Chemical structure of 3pY and the ESI mass spectrum data.

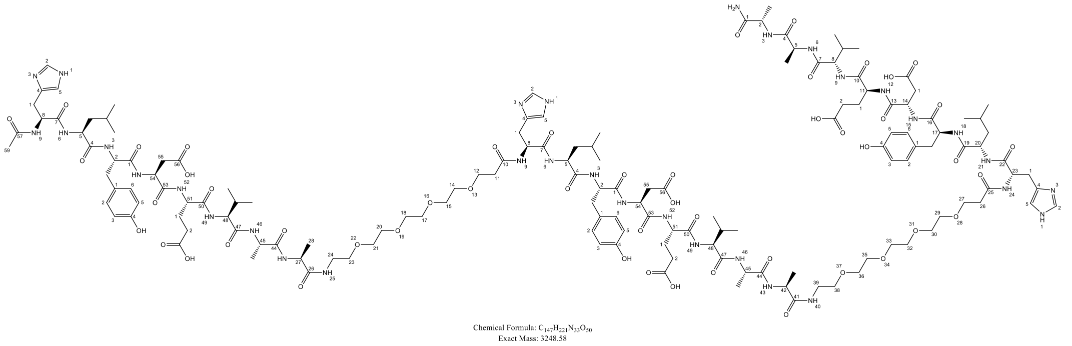

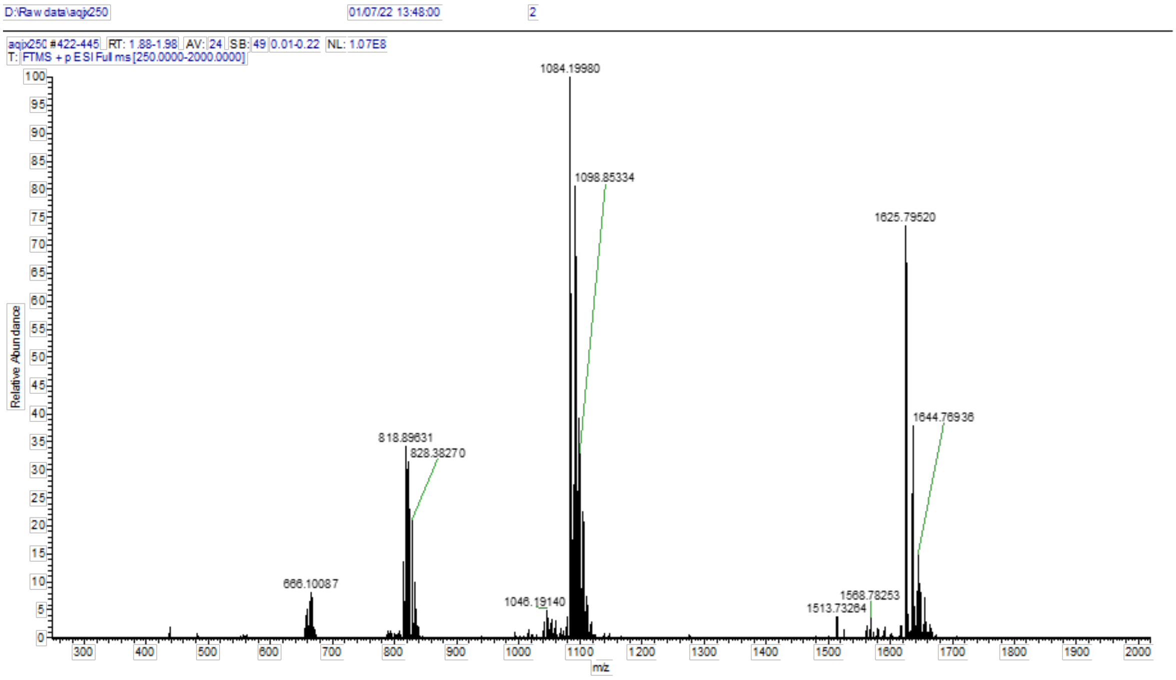

Molecular formula :	$C_{147}H_{221}N_{33}O_{50}$
Experimental Mass [M+2H]$^{2+}$, [M+3H]$^{3+}$, [M+4H]$^{4+}$:	1625.79520, 1084.19980, 813.40123
Theoretical Mass [M+2H]$^{2+}$, [M+3H]$^{3+}$, [M+4H]$^{4+}$:	1625.79702, 1084.20044, 813.40215
Error (ppm) :	-1.1, -0.6, -1.1

Figure S15. Chemical structure of 3Y and the ESI mass spectrum data.

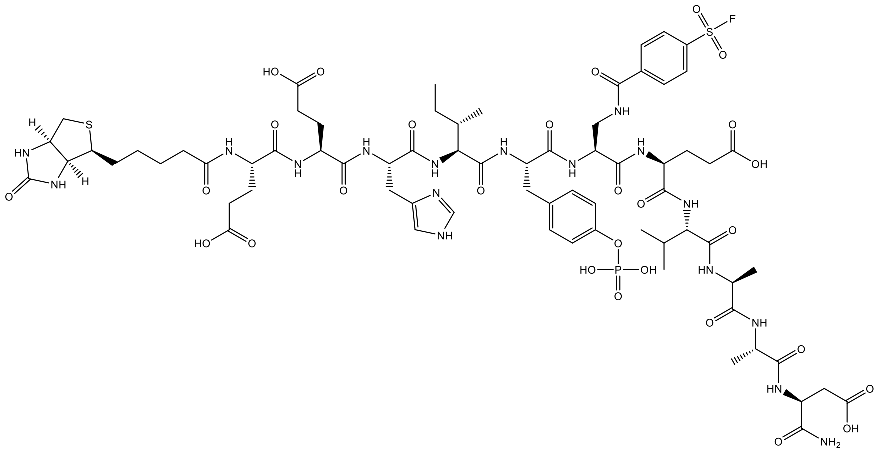

Chemical Formula: $C_{71}H_{99}FN_{17}O_{28}PS_2$
Exact Mass: 1751.60
Molecular Weight: 1752.76

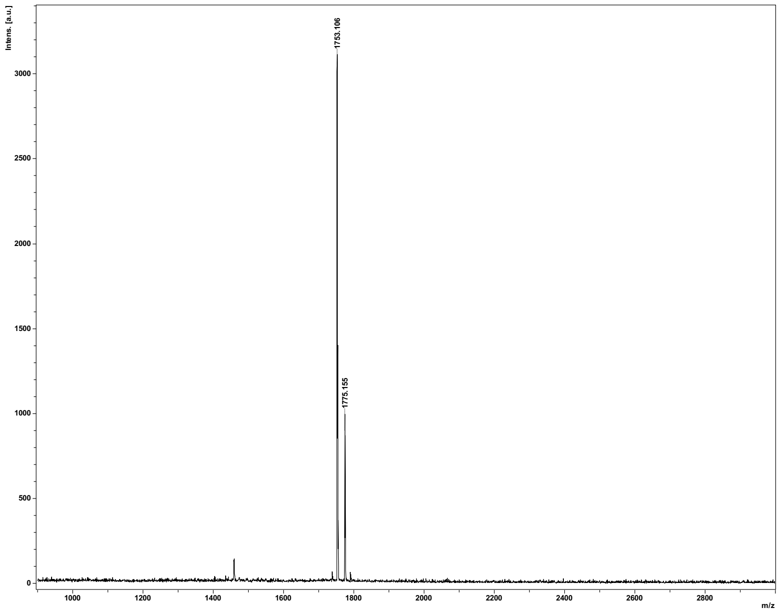

Figure S16. Chemical structure of p1 and the mass spectrum data by MALDI-TOF.

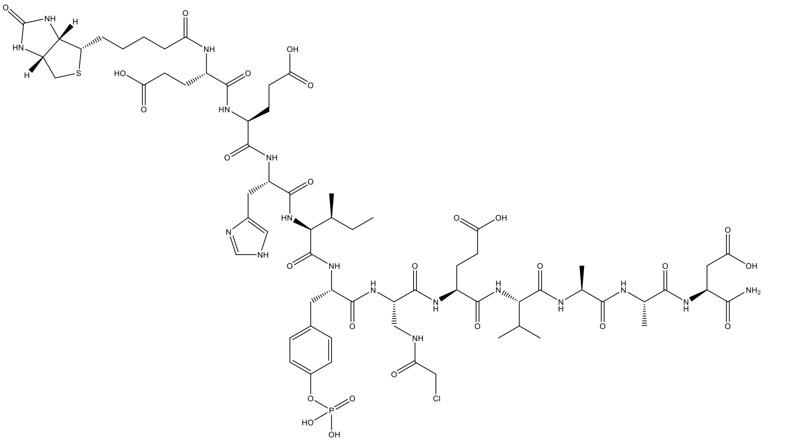

Chemical Formula: $C_{66}H_{97}ClN_{17}O_{26}PS$
Exact Mass: 1641.59
Molecular Weight: 1643.08

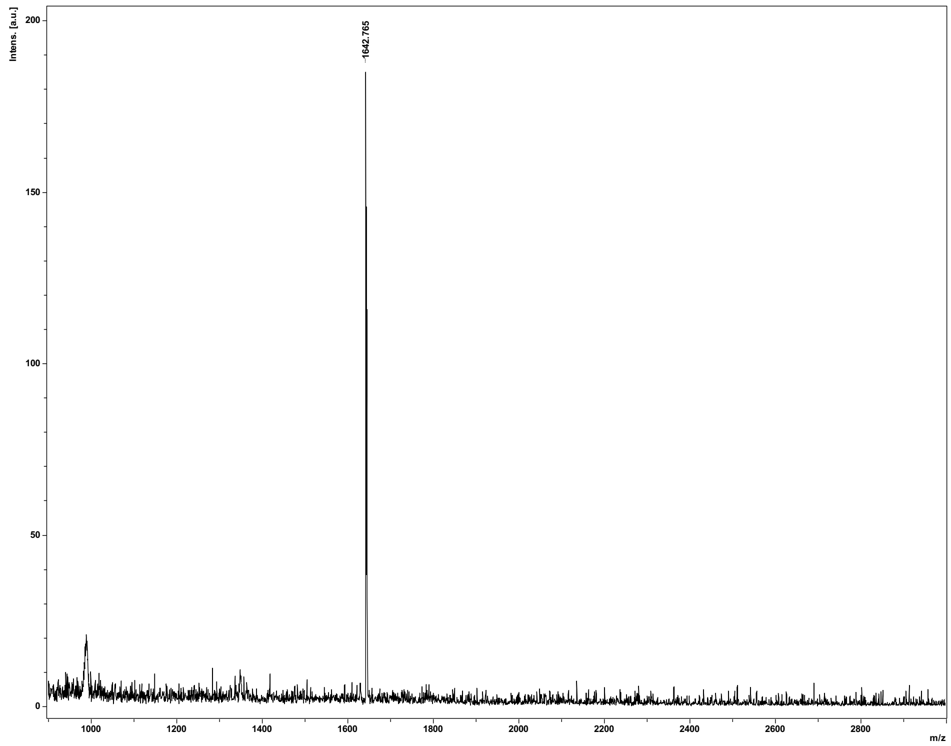

Figure S17. Chemical structure of p2 and the mass spectrum data by MALDI-TOF.

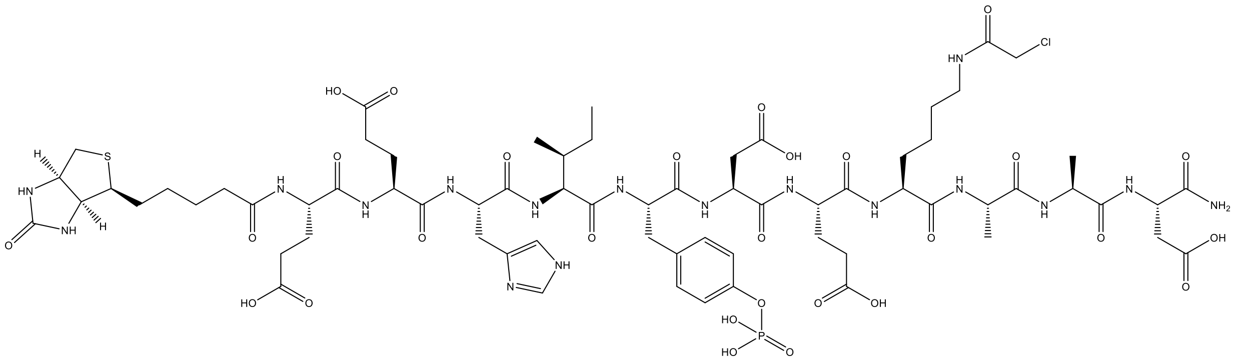

Chemical Formula: $C_{68}H_{99}ClN_{17}O_{28}PS$
Exact Mass: 1699.60
Molecular Weight: 1701.11

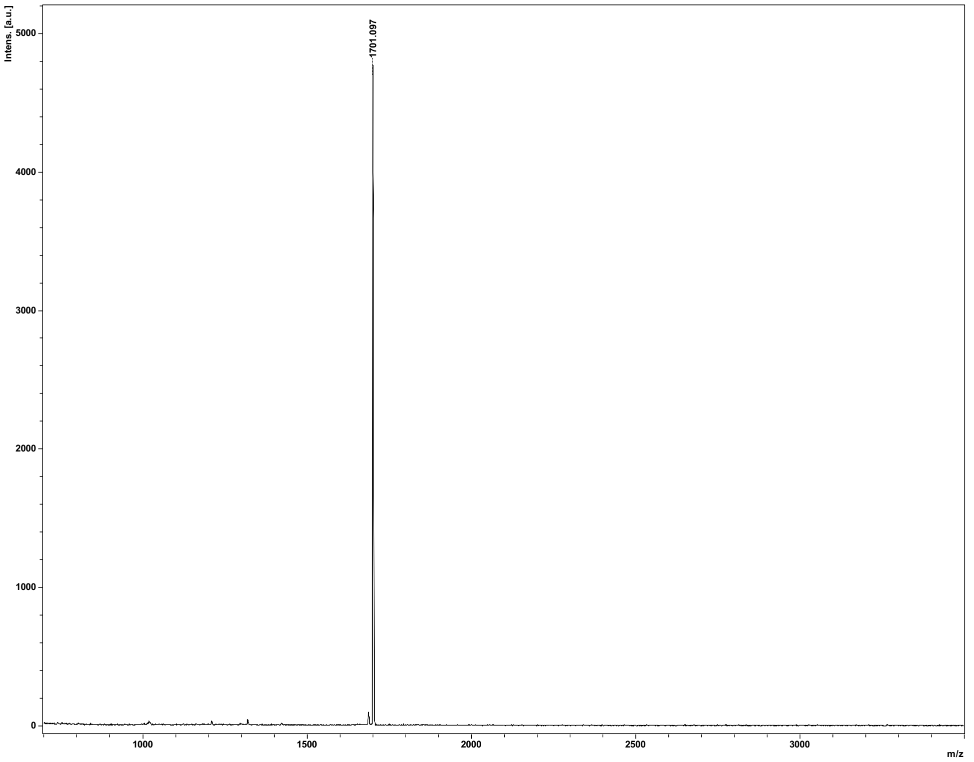

Figure S18. Chemical structure of p3 and the mass spectrum data by MALDI-TOF.

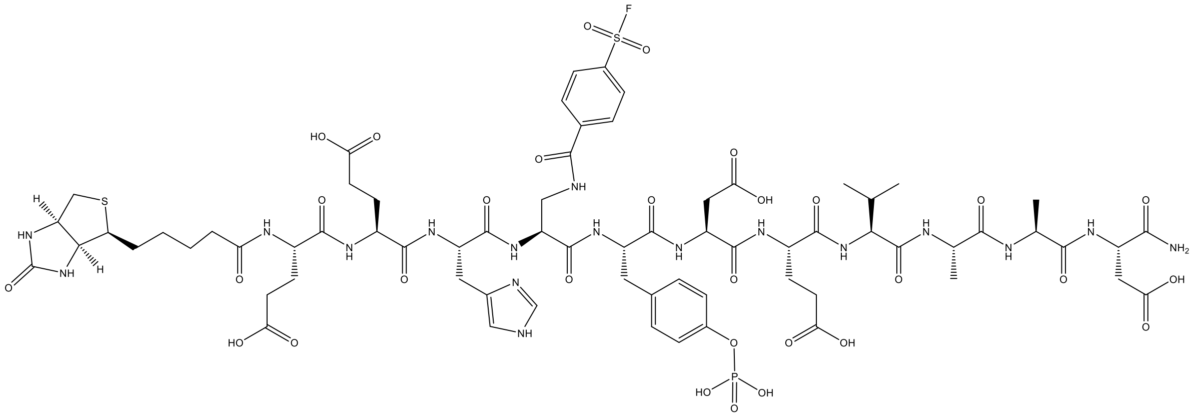

Chemical Formula: $C_{69}H_{93}FN_{17}O_{30}PS_2$
Exact Mass: 1753.54
Molecular Weight: 1754.68

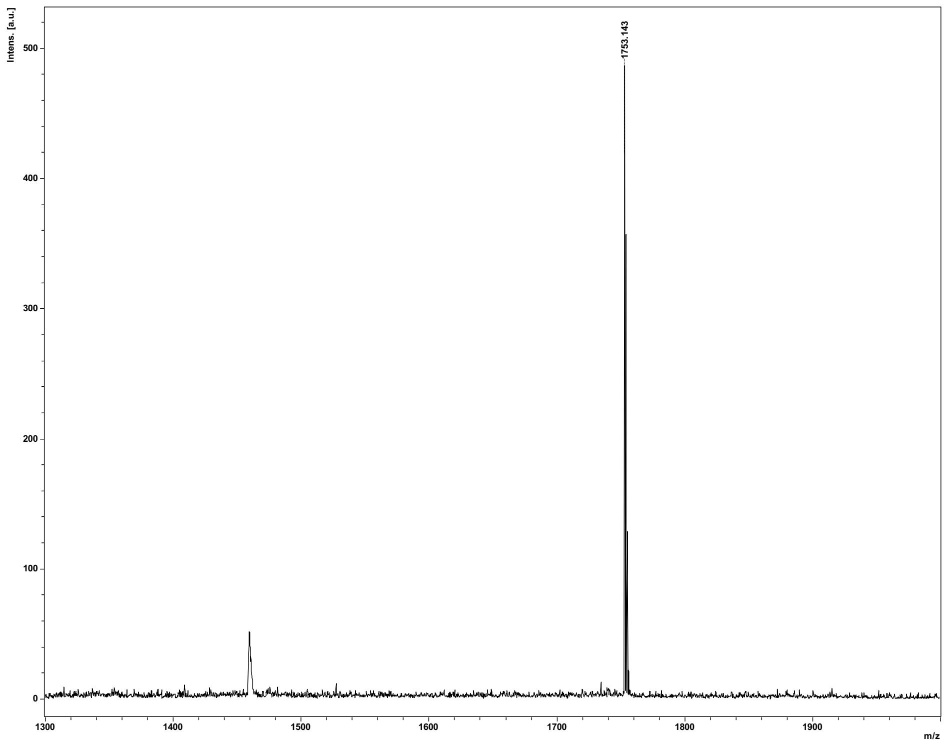

Figure S19. Chemical structure of p4 and the mass spectrum data by MALDI-TOF.

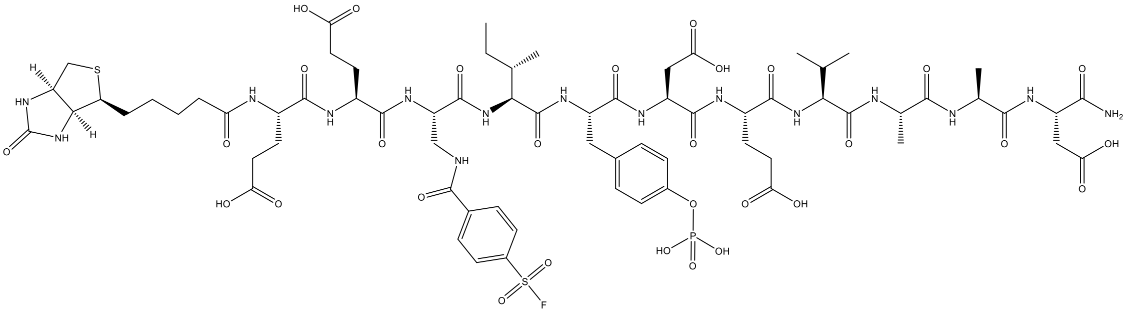

Chemical Formula: $C_{69}H_{97}FN_{15}O_{30}PS_2$
Exact Mass: 1729.57
Molecular Weight: 1730.70

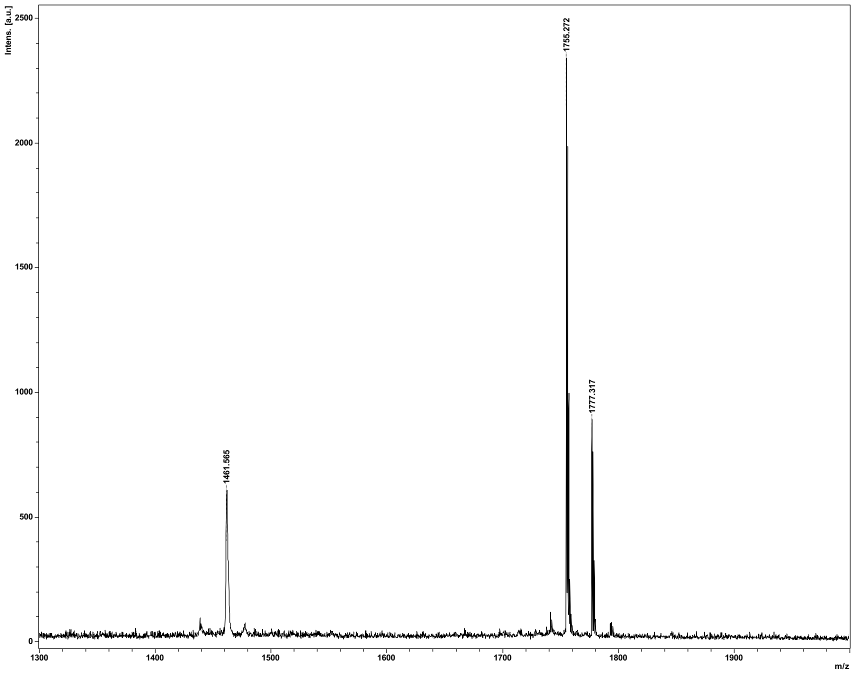

Figure S20. Chemical structure of p5 and the mass spectrum data by MALDI-TOF.

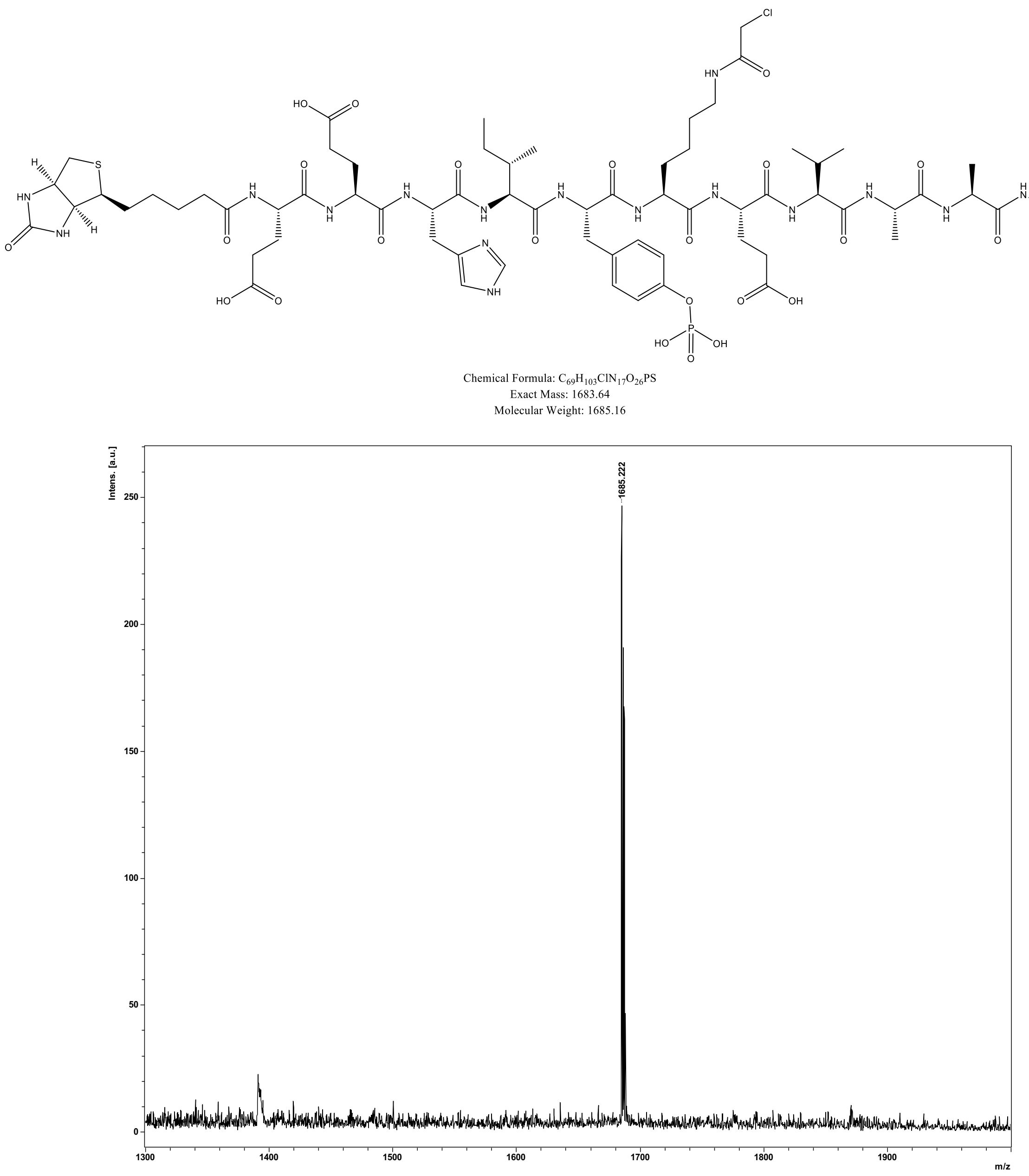

Figure S21. Chemical structure of p6 and the mass spectrum data by MALDI-TOF.

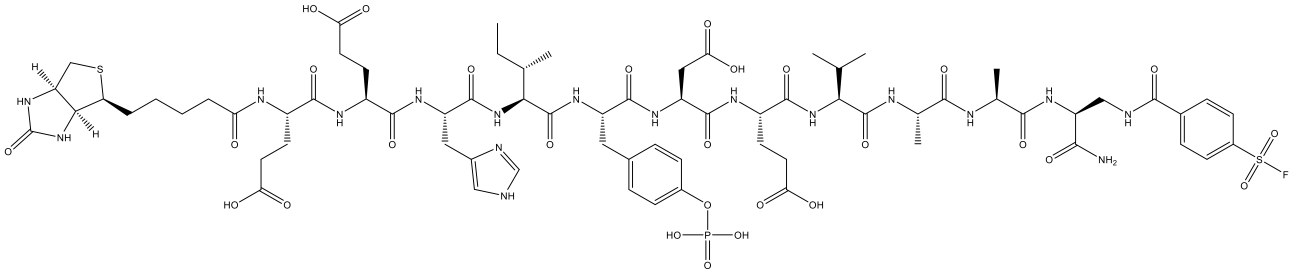

Chemical Formula: $C_{71}H_{99}FN_{17}O_{28}PS_2$
Exact Mass: 1751.60
Molecular Weight: 1752.76

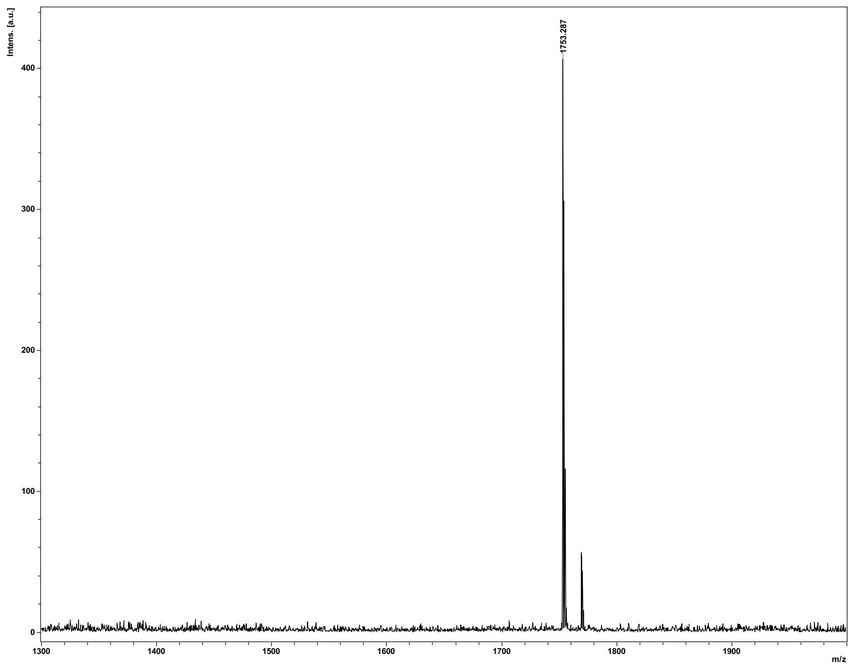

Figure S22. Chemical structure of p7 and the mass spectrum data by MALDI-TOF.

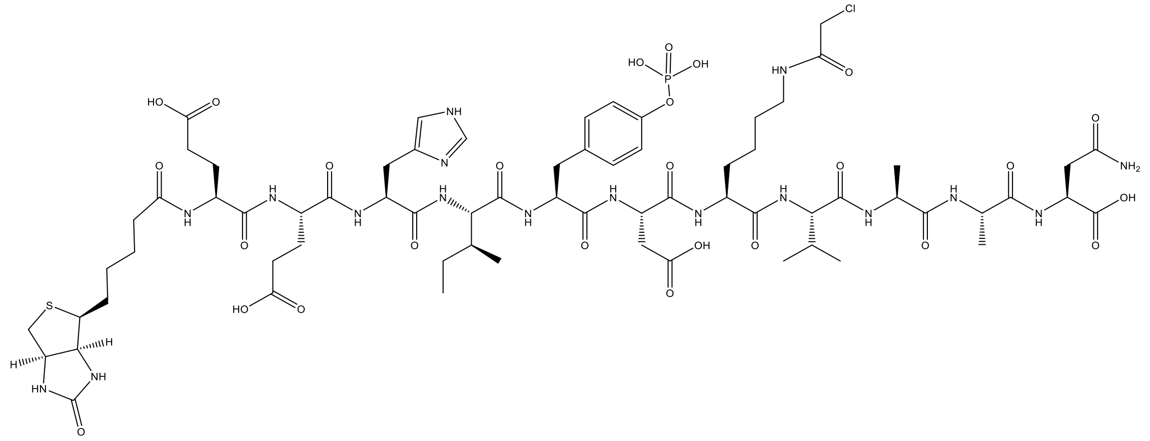

Chemical Formula: $C_{68}H_{101}ClN_{17}O_{26}PS$
Exact Mass: 1669.63
Molecular Weight: 1671.13

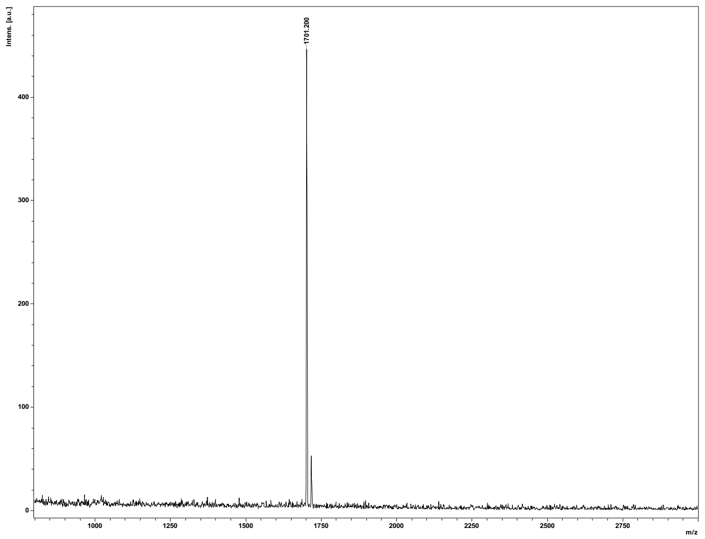

Figure S23. Chemical structure of p8 and the mass spectrum data by MALDI-TOF.

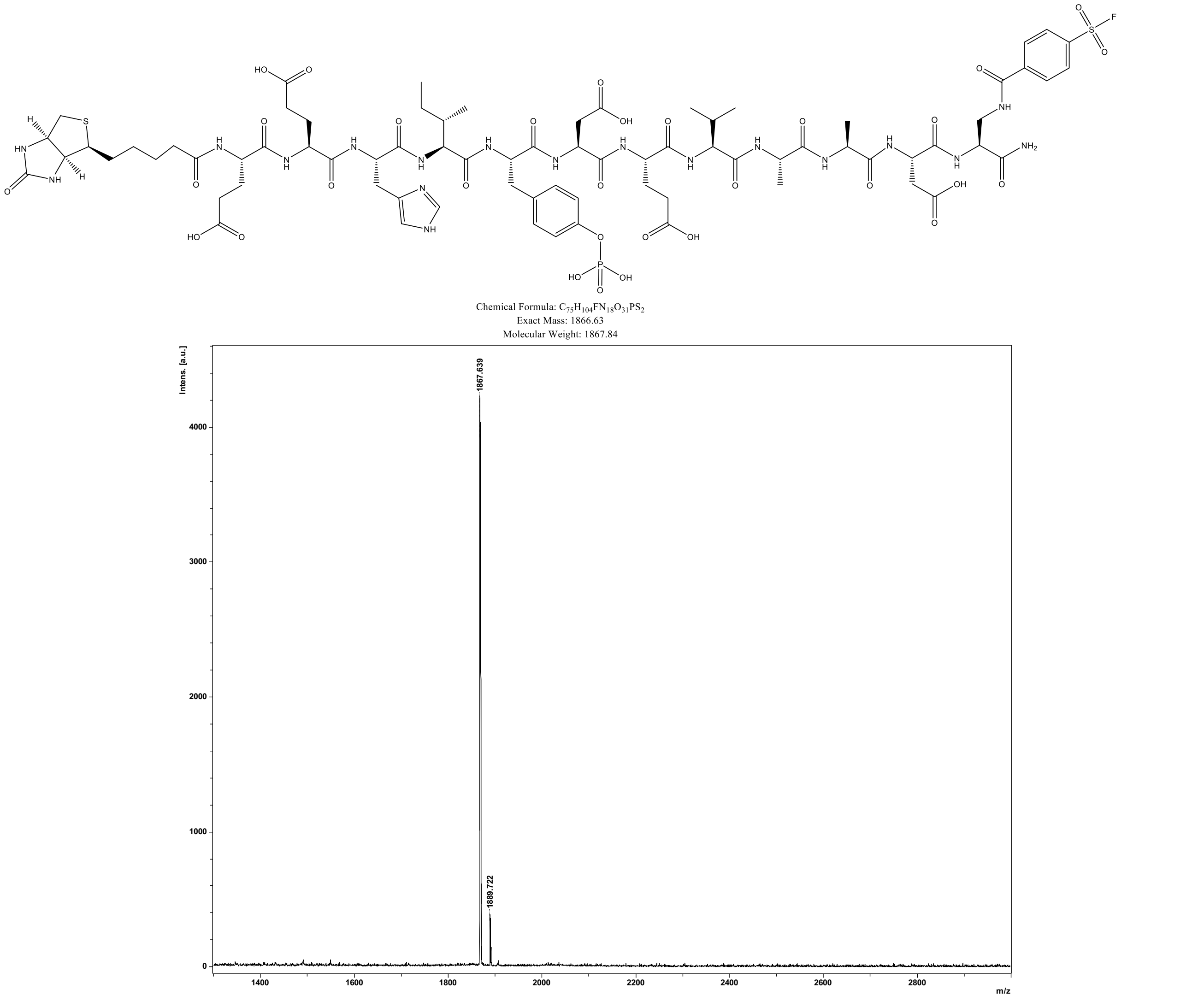

Figure S24. Chemical structure of p9 and the mass spectrum data by MALDI-TOF.

References

1. Chen, C.; Liang, Z.; Zhou, B.; Li, X.; Liu, C.; Ip, N. Y.; Qu, J. Y. In Vivo Near-Infrared Two-Photon Imaging of Amyloid Plaques in Deep Brain of Alzheimer's Disease Mouse Model. *ACS Chem. Neurosci.* **2018**, *9*, 3128–3136.

2. Wang, R.; Leung, P. Y. M.; Huang, F.; Tang, Q.; Kaneko, T.; Huang, M.; Li, Z.; Li, S. S. C.; Wang, Y.; Xia, J. Reverse Binding Mode of Phosphotyrosine Peptides with SH2 Protein. *Biochemistry* **2018**, *57*, 5257–5269.

3. Banjade, S.; Rosen, M. K. Phase transitions of multivalent proteins can promote clustering of membrane receptors. *Elife* **2014**, *3*, e04123.
